# Type 2 innate immunity drives distinct neonatal immune profile conducive for heart regeneration

**DOI:** 10.7150/thno.67515

**Published:** 2022-01-01

**Authors:** Francis M. Chen, Joyce KY Tse, Leigang Jin, Chui Yiu Bamboo Chook, Fung Ping Leung, Gary Tse, Connie W. Woo, Aimin Xu, Ajay Chawla, Xiao Yu Tian, Ting-Fung Chan, Wing Tak Wong

**Affiliations:** 1 School of Life Sciences, Faculty of Science, The Chinese University of Hong Kong, Hong Kong, China.; 2 State Key Laboratory of Pharmaceutical Biotechnology, The University of Hong Kong, Hong Kong, China.; 3 Department of Pharmacology and Pharmacy, The University of Hong Kong, Hong Kong, China.; 4 Tianjin Key Laboratory of Ionic-Molecular Function of Cardiovascular Disease, Department of Cardiology, Tianjin Institute of Cardiology, Second Hospital of Tianjin Medical University, Tianjin, China.; 5 Kent and Medway Medical School, Canterbury, United Kingdom.; 6 Department of Medicine, Li Ka Shing Faculty of Medicine, The University of Hong Kong, Hong Kong, China.; 7 Merck Research Labs, SAF-803, South San Francisco, CA, USA.; 8 School of Biomedical Sciences, The Chinese University of Hong Kong, Hong Kong, China.; 9 State Key Laboratory of Agrobiotechnology, The Chinese University of Hong Kong, Hong Kong, China.

**Keywords:** IL-4, IL-13, neonatal heart regeneration, T_H_2 immunity, alternatively activated macrophages, left anterior descending coronary artery ligation

## Abstract

**Aims:** Neonatal immunity is functionally immature and skewed towards a T_H_2-driven, anti-inflammatory profile. This neonatal immunotolerance is partly driven by the type 2 cytokines: interleukin-4 (IL-4) and interleukin-13 (IL-13). Studies on neonatal cardiac regeneration reveal the beneficial role of an anti-inflammatory response in restoring cardiac function after injury. However, the role of an imbalanced immune repertoire observed in neonates on tissue regeneration is poorly understood; specifically, whether IL-4 and IL-13 actively modulate neonatal immunity during cardiac injury.

**Methods and results:** Neonatal mice lacking IL-4 and IL-13 (DKOs) examined at 2 days after birth exhibited reduced anti-inflammatory immune populations with basal cardiac immune populations like adult mice. Examination of neonates lacking IL-4 and IL-13 at 2 days post cardiac ischemic injury, induced on the second day after birth, showed impaired cardiac function compared to their control counterparts. Treatment with either IL-4 or IL-13 cytokine during injury restored both cardiac function and immune population profiles in knockout mice. Examination of IL-4/IL-13 downstream pathways revealed the role of STAT6 in mediating the regenerative response in neonatal hearts. As IL-4/IL-13 drives polarization of alternatively activated macrophages, we also examined the role of IL-4/IL-13 signaling within the myeloid compartment during neonatal cardiac regeneration. Injury of IL-4Rα myeloid specific knockout neonates 2 days after birth revealed that loss of IL-4/IL-13 signaling in macrophages alone was sufficient to impair cardiac regeneration.

**Conclusions:** Our results confirm that the T_H_2 cytokines: IL-4 and IL-13, which skews neonatal immunity to a T_H_2 profile, are necessary for maintaining and mediating an anti-inflammatory response in the neonatal heart, in part through the activation of alternatively activated macrophages, thereby permitting a niche conducive for regeneration.

## Introduction

Ischemic heart disease is the leading cause of mortality and morbidity worldwide 1. Ischemia-induced injury leads to deterioration in cardiac function and increased fibrosis as adult mammalian cardiomyocytes are mitotically senescent, thus unable to proliferate in sufficient numbers to successfully resolve cardiac injury 2. However, neonatal mammalian cardiomyocytes have been shown to fully engage in the regenerative process because of a mitotically active cardiomyocyte population 3-5. Further studies mimicking ischemia in the neonatal heart through the ligation of the left anterior descending (LAD) coronary artery have identified the important roles of immune populations in the cardiac niche such as macrophages contributing to the regenerative process 6-8. While studies have indicated the importance of immunity in modulating successful functional regeneration in neonatal cardiac tissue, the effects of the neonate's immature immune system, distinct from adults, in regulating regeneration is relatively unexplored.

The mammalian neonatal immune niche was initially characterized as immunodeficient due to the weak responses exhibited to both bacterial infection and poor vaccine outcomes 9. However, increasing evidence indicates that neonatal immune systems are capable of mounting appropriate molecular and transcriptional immune responses, but skew towards upregulating T_H_2 driven programs that dampen traditionally pro-inflammatory responses 9-12. Contemporary studies of neonatal immunity reveal an immune system prioritized toward immunosuppressive programs driven primarily by the classical T_H_2 cytokine IL-4, and to a lesser degree IL-13 11, 13-17. The ontogeny of neonatal immunity is driven in part by the need to maintain fetomaternal tolerance, an immune-privileged state between mother and child, while allowing for the development of beneficial populations of the microbiome 9, 18. While various neonatal immune populations ranging from Tregs to macrophage populations have been shown to resolve injury in the neonatal heart, whether the underlying T_H_2 immune preference in neonates, driven largely by the T_H_2 cytokines: IL-4 and IL-13, affects regeneration outcomes in injured cardiac tissue is unknown 6, 19-21.

IL-4 and IL-13 are pleiotropic cytokines classically associated with T_H_2 cell maturation, the emergence of allergic and asthmatic pathologies, and the regulation of immune responses to helminth infection 22, 23. The roles of both IL-4 and IL-13 in tissue regeneration have been recently demonstrated, both in modulating immune and stem cell populations in the injured niche 24-27. In each case, IL-4/IL-13-signaled cell types within the injured tissue to regulate sectors within the niche, either the physical characteristics or the cellular components to facilitate the return to homeostatic functionality 28-30. Furthermore, experiments performed in adult murine hearts coupled with transcriptomic analysis of neonatal hearts have indicated the reparative functions of IL-4 and IL-13 31, 32. Other studies have shown the role of IL-4 and IL-13 in regulating cardiomyocyte functionality, while also regulating alternatively activated macrophages in a variety of tissue types 33-35. However, to the best of our knowledge, none has characterized the role of IL-4 and IL-13 directly on immune populations in the regenerating neonatal heart.

Due to the prevalence of both IL-4 and IL-13 in regulating T_H_2 neonatal immunity, we sought to identify the role of both cytokines in regulating cardiac regeneration in the neonate 36, 37. In this article, we present evidence that the type 2 immune responses regulated by IL-4/IL-13 stimulate cardiac regeneration in the neonate in the presence of ischemic injury. Moreover, loss of IL-4 and IL-13 signaling via the receptor of both cytokines, IL-4Rα in myeloid cells alone resulted in the loss of regenerative capacity in injured neonatal cardiac tissue.

## Methods

### Experimental Animals

All animal experiments were performed with the approval and supervision of the Chinese University of Hong Kong (CUHK) Animal Experimentation Ethics Committee and the University Laboratory Animal Services Center (LASEC), License#: (18-728) in DH/SHS/8/21 Pt.14. Timed-pregnancies of strains: *BALB/c* (CON)*, IL-4^-/-^/IL-13^-/-^* (DKO), *IL-4Rα^ff^* (CRE^-^), and *IL-4Rα^ff^xLyz2^Cre^* (CRE^+^) were used to age-match pups at P2. Both DKO, CRE^-^, and CRE^+^ strains were given as gifts by the lab of Dr. Ajay Chawla (Merck, South San Francisco, CA). *BALB/c* control strain was purchased and bred from LASEC at the Chinese University of Hong Kong. DKO mice were bred on a *BALB/c* background, while CRE^-^ and CRE^+^ mice were bred on a *C57B/6* background. The day of birth is considered P1. Mice were housed in a temperature controlled room with 12-hour light/dark cycle in the Laboratory Animal Services Center (LASEC) with free access to water and food *ad libitum*. Sex of the neonate was not determined for experiments herein described. Age and number of mice used are indicated per figure.

### Neonatal Myocardial Infarction Model (MI)

MI surgeries were performed on P2 neonatal mice (*BALB/c*, *IL-4^-/-^/IL-13^-/-^*,*IL-4Rα^ff^*, and *IL-4Rα^ff^xLyz2^Cre^* strains, Laboratory Animal Services Center), as previously described 38, 39. Briefly, neonatal mice at 2 days after birth were placed in a sealed 1200 cm^3^ chamber with approximately 5% isoflurane until cessation of movement. Neonate was removed from chamber and placed on ice-filled nitrile glove to induce hypothermia-induced anesthetization on ice. Povidone-iodine and 70% isopropanol were used as antiseptics prior to surgery. Lateral thoracotomy was performed at the fourth intercostal space. A tapered needle attached to a 10-0 prolene non-absorbable suture (Surgical Specialties, Wyomissing, PA) was passed below the left anterior descending coronary artery and ligated to induce ischemic infarction. Total surgical time of neonate under hypothermia-induced anesthetization did not exceed five minutes. After surgery, incisions were sutured with an 8-0 nonabsorbable prolene suture (Surgical Specialties). Neonates recovered under a heat lamp for 15 minutes with maternal bedding before returning to mother for rearing. All surgical tools were sterilized by open flame and ethanol prior and during use. Neonates were subsequently euthanized by decapitation prior to tissue extraction.

### Neonatal treatment of cytokines/drugs via intraperitoneal injection during MI model

Mouse IL-4 (Peprotech, Rocky Hill, NJ) and IL-13 (Peprotech) were injected at 10 mg/kg concentrations via intraperitoneal injection of the neonatal mice immediately following LAD ligation surgery as described previously 31. AS1517499 (Millipore-Sigma, Burlington, MA) was injected at 10 mg/kg concentrations one hour prior to LAD ligation surgery and was injected at 24 hour intervals until sample harvest for 2 days post injury (DPI) flow cytometric experiments or 19 DPI following echocardiography assays for morphological analysis 40. Mice at time points beyond 7 days were euthanized with CO_2_ asphyxiation and cervical dislocation. Mice between 2 days after birth to 7 days were euthanized with decapitation.

### Transthoracic Echocardiography

Cardiac function and heart dimensions were evaluated by 2-dimensional echocardiography using a Visual Sonics Vevo 2100 Ultrasound (FUJIFILM Visual Sonics, Toronto, Canada) on anesthetized mice. Mice placed on a heated 37 °C dock and anesthetized with a nose cone releasing 0.5-1% isoflurane at a constant rate. M-mode tracings were used to measure anterior and posterior wall thicknesses at both end diastole and systole. Left ventricular internal diameter was measured as the largest anteroposterior diameter in either diastole (LVID;d) or systole (LVID;s). A single observer blinded to mouse experimental groups performed echocardiography and data analysis.

### Extended Methods

An extended compendium of detailed methodology and materials are available in the Supplementary Material. A list of materials in the form of a Major Resources Table is also available in the Supplemental Materials under Supplemental Table 1.

### Data Availability

All supporting data within this article and its supplementary files are available. RNA-seq data sets generated can be accessed at the NCBI public repository using the accession number PRJNA730354. Additional information such as qPCR primer sequences, etc. will be available upon request to the corresponding authors.

## Results

### IL-4 and IL-13 maintain the neonatal cardiac immune niche distinct from adults

**Figure 1:**
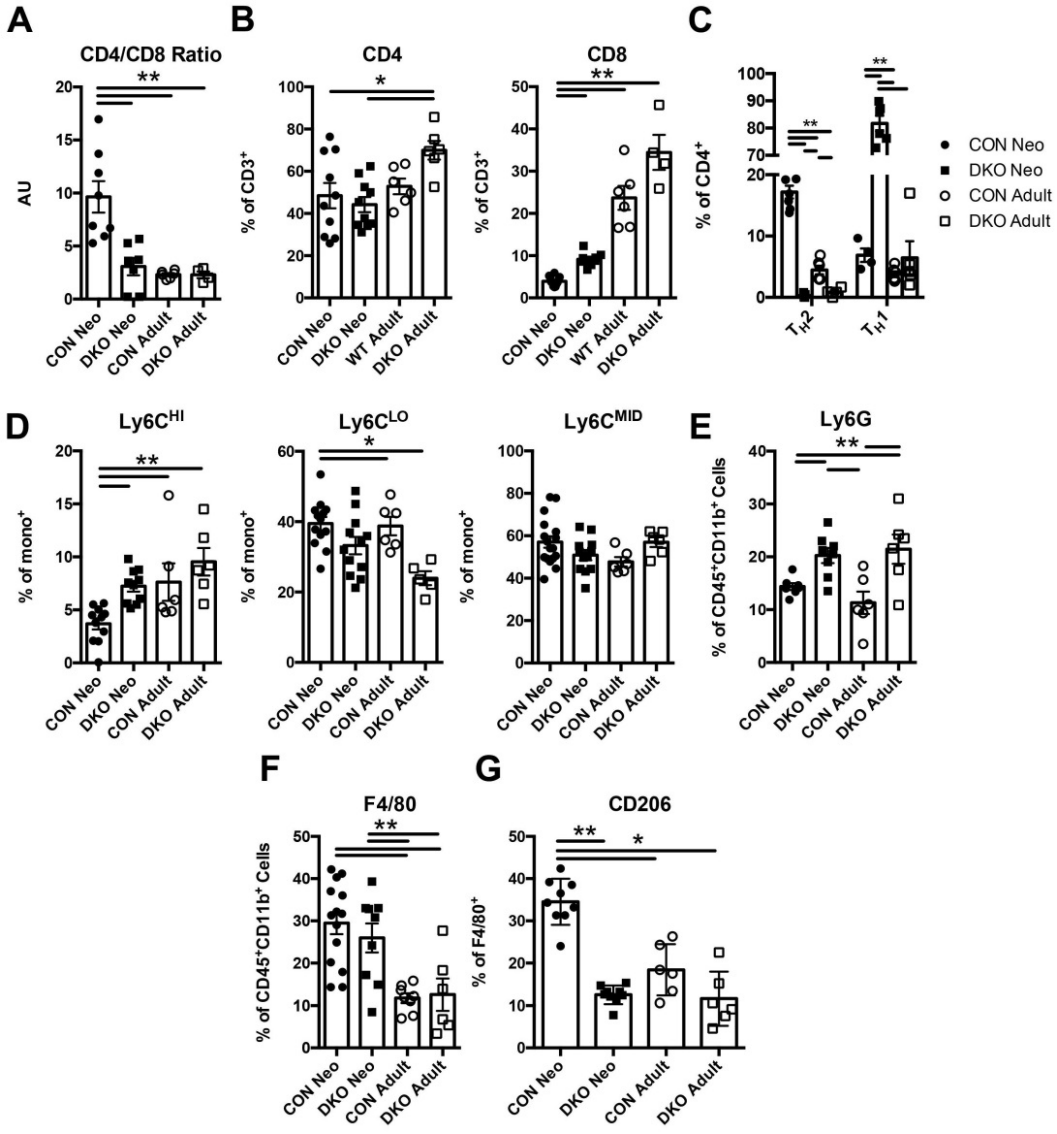
** IL-4/IL-13 maintains neonatal immune landscape distinct to adult immune populations in the heart.** P2 (2 days after birth) neonatal and adult (10 week old) *BALB/c* control (CON) and *IL-4^-/-^/IL-13^-/-^* (DKO) hearts were isolated to determine immune populations using flow cytometry. *(A)* CD4/CD8 Ratios (n = 5-8). *(B)* CD4^+^, CD8^+^ populations (n = 5-8). *(C)* T_H_1 and T_H_2 populations of CD4^+^ T-cells (n = 5-6); [**P < 0.01 , T_H_1-all groups significant except DKO neo vs DKO adult]. *(D)* Ly6C^HI/LO/MID^ monocyte populations (n = 6-12). *(E)* Ly6G^+^ neutrophil populations (n = 6-12). *(F)* F4/80^+^ macrophage populations (n = 6-12). *(G)* Alternatively activated F4/80^+^CD206^+^ macrophage populations (n = 6-8). Data are presented as mean±SEM. *P < 0.05, **P < 0.01. by two-way ANOVA followed by Sidak's multiple comparisons test.

To ascertain whether IL-4 and IL-13 regulates neonatal immune preference in the heart, we first examined the composition of immune cells in both uninjured *BALB/C* (CON) and *IL-4^-/-^/IL-13^-/-^* (DKO) neonatal cardiac tissue compared to adult. Hearts from P2 (2 days after birth) neonates and 10-week-old CON and DKO mice were isolated; their cellular populations were identified using flow cytometry (Figure S1A). Characterization of immune populations such as T-cells, monocytes, neutrophils, and macrophages revealed a difference between the basal neonatal and adult immune populations, with the neonate preferring anti-inflammatory immune populations compared to the more balanced immune landscape observed in adult hearts (Figure 1A-G). Examination of CD4^+^ and CD8^+^ T-cell populations revealed a larger population of CD4^+^ T-cells compared to CD8^+^ T-cells in neonatal hearts whereas loss of IL-4 and IL-13 increased CD8^+^ T-cells (Figure 1B). The increase in CD8^+^ T-cells observed in injured DKO neonatal hearts recapitulates studies describing IL-4's role in repressing CD8^+^ T-cell expansion in the neonate 15. Moreover, the ratio of CD4^+^ to CD8^+^ T-cells, a representative indicator of immune health and the prevalence of CD4^+^ to CD8^+^ T-cells, indicated that loss of IL-4/IL-13 resulted in a lower CD4/CD8 ratio compared to neonatal CON hearts (Figure 1A). This reduction of the CD4/CD8 ratio in DKO neonatal hearts mirrored those of adult CON hearts. Furthermore, much of the CD4^+^ T-cells in DKO neonates were T_H_1, using IFN-γ as a marker, compared to control neonatal hearts which preferred CD4^+^ T_H_2 populations, using IL-4 as a marker (Figure 1C). Examination of monocyte populations indicated a preference for anti-inflammatory Ly6C^LO^ monocyte populations compared to Ly6C^HI^ pro-inflammatory monocytes in CON neonates (Figure 1D). Neutrophil populations were increased in both neonatal and adult DKO populations compared to CON (Figure 1E). Finally, while both the percentage of CON and DKO neonatal macrophages were greater than those in adult hearts, a larger fraction of CON neonatal macrophages was alternatively activated (Figure 1F and G). The percentage of DKO neonatal macrophages which were polarized to a M2 state, using CD206 as a marker, were similar to both adult CON and DKO hearts (Figure 1G). These M2 macrophages, or alternatively activated macrophages, have been identified for their reparative functions after injury in a variety of tissue types 41.

### IL-4 and IL-13 are required for the regeneration of neonatal hearts after ischemic injury

**Figure 2:**
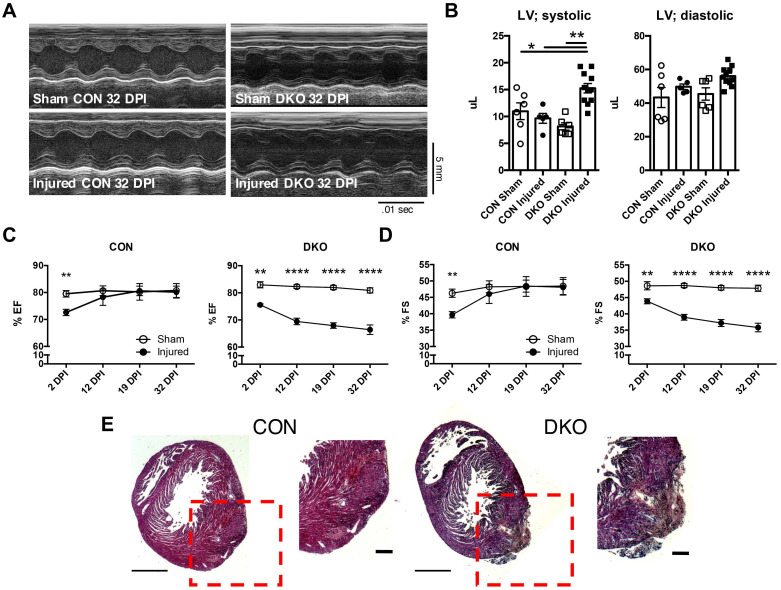
** Loss of IL-4 and IL-13 impairs regeneration following injury in neonatal hearts.** P2 neonatal hearts underwent left anterior descending (LAD) coronary artery ligation and underwent echocardiography across 2, 12, 19, and 32 days post injury (DPI). *(A)* Representative echocardiograms of CON and DKO neonatal hearts at 32 DPI. *(B)* Increased left ventricular systolic volume (LV; systolic) in injured DKO hearts indicating hypertrophic response, but no change in left ventricular diastolic volume (LV; diastolic) (n = 6-10). *(C and D)* Functionality of CON and DKO neonatal hearts following ischemic injury via ejection fraction and fractional shortening (n = 8-10). *(E)* Representative hematoxylin & eosin staining of WT and DKO injured heart micrographs at 12 DPI (n = 5). Scale Bar: 1 mm, 200 μm (Red inset). Data are presented as mean±SEM. **P < 0.01, ****P < 0.0001 by two-way ANOVA followed by Sidak's multiple comparisons test.

To address the functional role of IL-4 and IL-13 in neonatal hearts after injury, we ligated the left anterior descending (LAD) coronary artery to mimic an infarct event in neonatal control and DKO mice 3, 38. Cardiac function was assessed using echocardiography at 2, 12, 19, and 32 DPI (Figure 2A). Both fractional shortening and ejection fraction of CON neonatal hearts returned to sham control levels after the initial reduction observed at 2DPI (Figure 2C and D). However, DKO neonatal mice saw a progressive deterioration in cardiac functionality over the same period (Figure 2C and D). An examination of the systolic and diastolic left ventricular volume indicated an enlarged left ventricle during systole in the DKO injured (Figure 2B). Further histological examination revealed extensive multicellular infiltration in DKO injured neonatal hearts compared to control injured neonatal hearts at 12 DPI, similar to injured cardiac tissue with immune infiltration observed by other groups (Figure 2E) 42, 43. As such, echocardiographic examination of both CON and DKO neonatal hearts after LAD coronary artery ligation revealed that loss of IL-4 and IL-13 delayed the functional recovery of the injured cardiac tissue.

### Anti-inflammatory populations are regulated by IL-4 and IL-13 to maintain a conducive environment for cardiac regeneration

**Figure 3:**
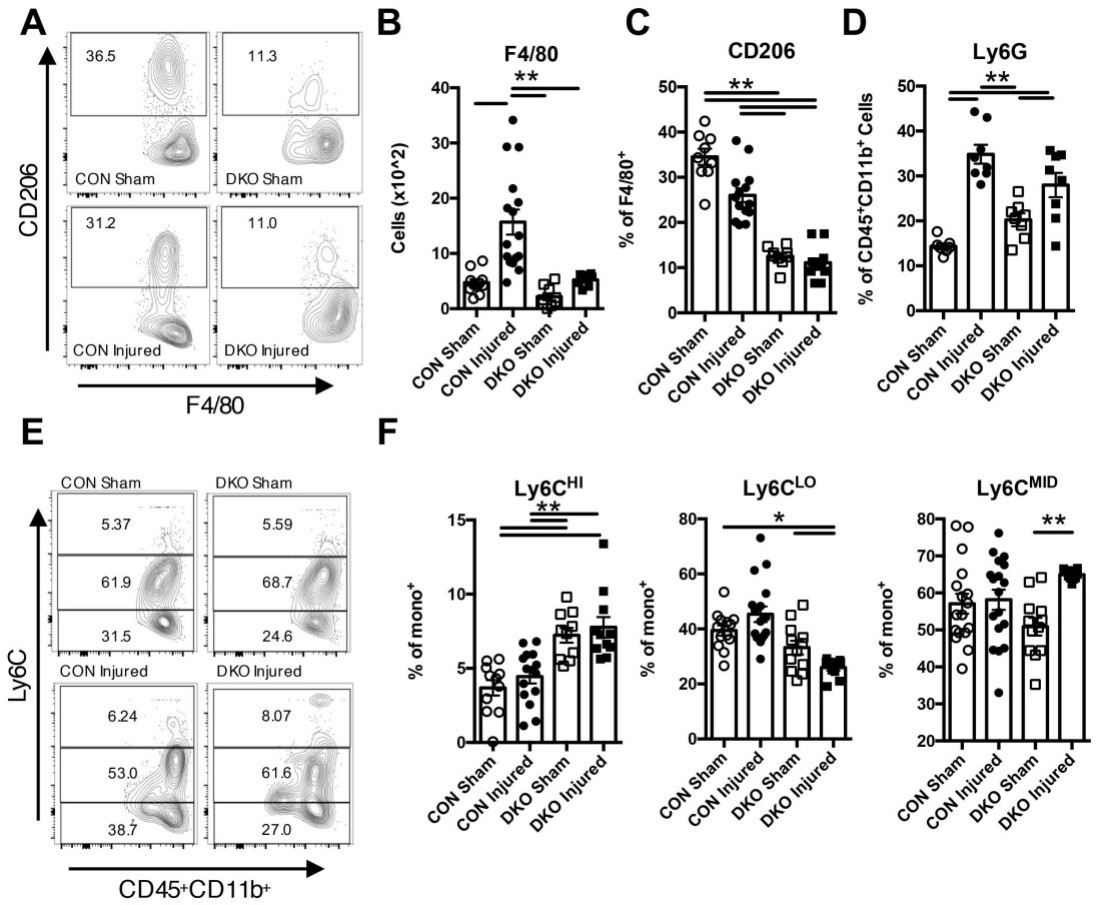
** IL-4/IL-13 signaling promoted anti-inflammatory response in neonatal heart regeneration.** P2 (2 days after birth) neonatal mice lacking IL-4 and IL-13 with corresponding controls underwent LAD ligation and harvested at 2 days post injury (DPI). Different immune populations were surveyed using flow cytometric analysis, specifically: F4/80 macrophages, M2 F4/80^+^CD206^+^ macrophages, Ly6G^+^ neutrophils, and Ly6C^HI/MID/LO^ monocytes. Ly6C^Hi^ monocytes defined as increasingly pro-inflammatory whereas Ly6C^LO^ being more anti-inflammatory in nature. *(A)* Representative flow plot of F4/80^+^CD206^+^ populations. *(B)* F4/80^+^ macrophage populations (n = 9-15). *(C)* F4/80^+^CD206^+^ populations (n = 10-12). *(D)* Ly6G^+^ neutrophil populations (n = 7-8). *(E)* Representative flow plots of Ly6C^HI/MID/LO^ monocyte populations. *(F)* Ly6C^HI/LO/MID^ monocyte populations (n = 9-12), *(G)* Data are presented as mean±SEM. * P < 0.05, **P < 0.01 by two-way ANOVA followed by Sidak's multiple comparisons test.

As DKO injured neonatal hearts exhibited clear signs of functional degeneration after injury, we examined how IL-4/IL-13 modulates the immune niche in the neonatal heart after ischemic injury. Initial time course characterization of CON injured neonatal hearts indicated significant changes in the population of various immune cell subtypes at 2 DPI (Figure S3). Thus, we chose to examine immune populations in both CON and DKO neonatal hearts 2 days after LAD coronary artery ligation. Examination of overall hematopoietic lineage cells indicated a clear increase in cell numbers after injury in CON neonatal hearts that was not present in DKO neonates (Figure S4A and B). CD3^+^ cells were greatly expanded in injured DKO hearts (Figure S4C), with a large proportion of those cells being CD8^+^ cytotoxic T-cells (Figure S5D and F) while there were no significant changes in CD4^+^ T-cells (Figure S5C and D). An examination of other immune populations such as macrophage populations indicated a significant increase response in CON neonates compared to DKO neonates (Figure 3A and B). The proportion of M2 macrophages, as represented by CD206 expressing F4/80^+^ macrophages, were significantly reduced in both DKO sham and DKO injured hearts (Figure 3A and C). Consequently, examination of monocyte numbers revealed three separate populations, with a majority of cells in both CON and DKO being Ly6C^MID^ (Figure 3E and F). Expression of Ly6C^HI^ and Ly6C^LO^ populations were distinctly bifurcated with DKO neonates preferring the former and CON neonates preferring the latter, specifically DKO neonates expressing a larger proportion of Ly6C^HI^ compared to CON and a lower proportion of Ly6C^LO^ monocytes (Figure 3E and F). Multiple sources have commented on the pro-inflammatory nature of Ly6C^HI^ circulating monocytes compared to the more anti-inflammatory Ly6C^LO^ monocyte population 44. Interestingly, examination of Ly6G neutrophil populations did not reveal a significant difference in response to injury from both CON and DKO hearts (Figure 3D).

### Both IL-4 and IL-13 are sufficient to maintain a conducive environment for cardiac regeneration, signaling discrete immune populations in the niche

**Figure 4:**
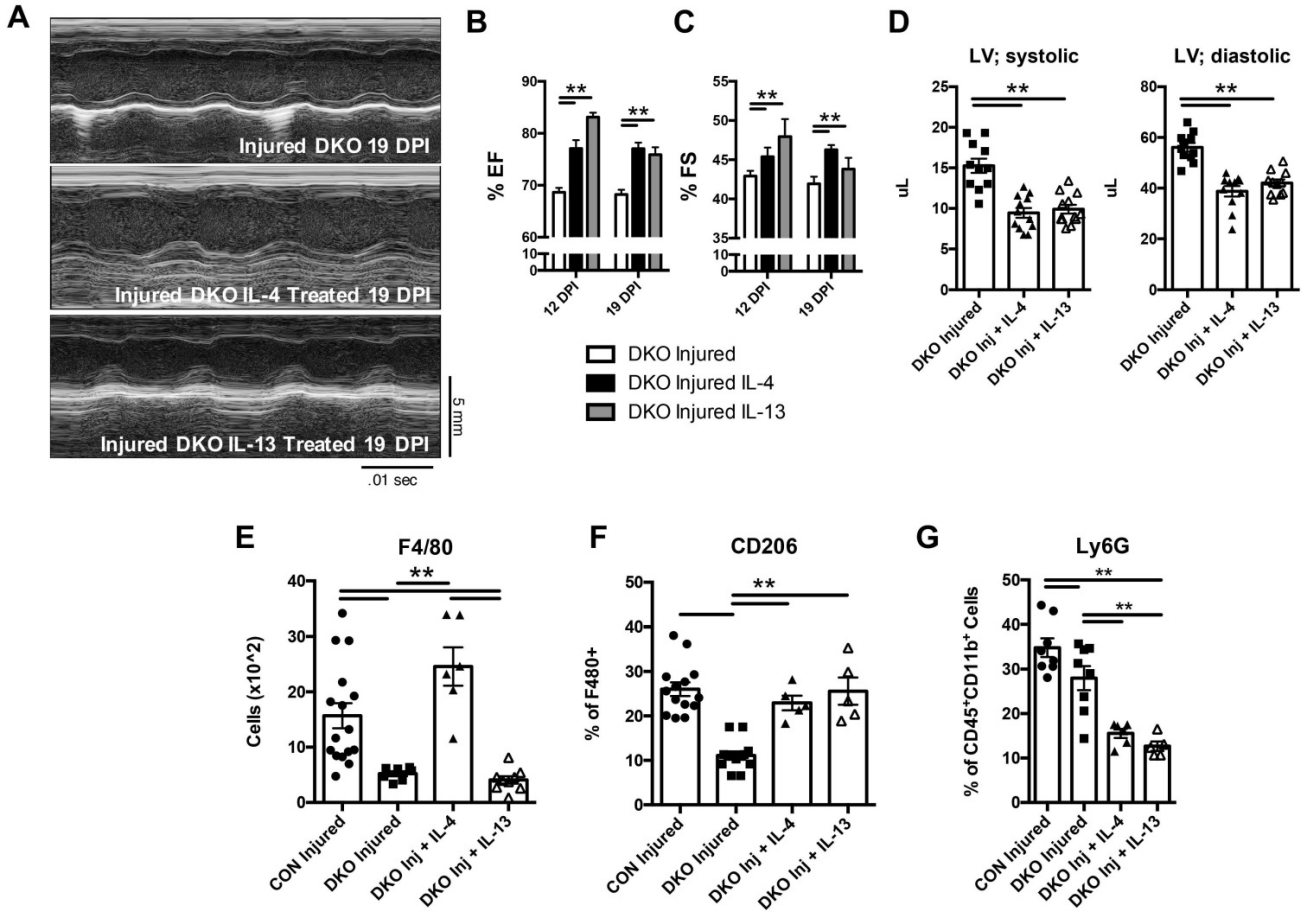
** IL-4 or IL-13 rescues cardiac regeneration defect observed in DKO neonates.** 2 day old neonates lacking IL-4 and IL-13 underwent LAD coronary ligation and injected intraperitoneally with either IL-4 or IL-13 immediately after. Heart samples were subsequently analyzed for the following characteristics: *(A)* Representative echocardiograms of DKO treated with IL-4/IL-13 at 19 DPI. *(B)* Ejection fraction (EF) (n = 8-10). *(C)* Fractional shortening (FS) (n = 8-10). *(D)* Left ventricular systolic and diastolic volumes (n = 11-12). *(E)* Absolute numbers of F480^+^ macrophages (n = 6-8). *(F)* CD206^+^ macrophages (n = 5-8). *(G)* Ly6g neutrophils (n = 6-8) after IL-4 or IL-13 treatment. Data are presented as mean±SEM. * P < 0.05, **P < 0.01. by one-way ANOVA followed by Sidak's multiple comparisons test for groups. Scale bar = 200 μm.

To gain an understanding on which cytokine was necessary for the maintenance of the T_H_2 mediated response in neonatal hearts and whether each cytokine played a discrete role in regulating immune populations, we injected separately, IL-4 and IL-13, in DKO neonatal mice immediately after injury. Echocardiography was subsequently performed after LAD coronary artery ligation at 12 and 19 DPI. Treatments of IL-4 and IL-13 independently restored cardiac function after injury compared to DKO (Figure 4A-D). Further examination of immune populations indicated a preference of each cytokine to upregulate anti-inflammatory states in different immune populations, indicating discrete functionality of each cytokine in neonatal hearts in response to injury. Specifically, IL-4 restored overall CD45^+^ hematopoietic cells in DKO compared to IL-13 (Figure S5A). Also, IL-4 had a greater impact in T-cell populations compared to IL-13. Use of IL-4 restored CD4^+^ preference over CD8^+^ T-cells in DKO while there were no significant changes in IL-13 treated DKO mice (Figure S5C and D). Although IL-13 treatment did not change T-cell populations in DKO neonates, it did restore the anti-inflammatory phenotype of monocyte and macrophage populations in DKO mice similar to IL-4 (Figure 4E and G, Figure S5E). Specifically, Ly6C^HI^ monocyte populations were reduced compared to Ly6C^LO^ monocyte in both IL-4 and IL-13 treated DKO injured mice (Figure S5E), and there was a restoration of alternatively activated macrophage populations shown by the increase in CD206^+^ macrophages (Figure 4E and F). The differences in treatment of IL-4 on hematopoetic stem cell and macrophage populations have been shown by other groups in other tissue types 28, 45. Interestingly, both IL-4 and IL-13 treatment significantly reduced the number of neutrophils compared to both control and DKO injured hearts (Figure 4G). These results indicate that while IL-4 and IL-13 act on different immune cell populations, both could functionally rescue the heart after injury by reducing the proportion of pro-inflammatory monocytes and increasing the proportion of alternatively activated macrophages.

### Transcriptional landscape indicate loss of IL-4 and IL-13 results in an increase in negative factors to cardiac regeneration

With a reduction in anti-inflammatory cells in DKO injured neonatal hearts, we sought to determine the transcriptional programs regulating the various extrinsic factors that could negatively impact cardiac regeneration without the expression of IL-4 and IL-13. Again, littermates were injured on the second day after birth and RNA were isolated and sequenced compared to sham littermate controls at 2 DPI. Characterization of sequenced data using principal component analysis indicated that similar experimental replicates of samples were closely clustered to each other, with CON and DKO neonates grouped in separate groupings (Figure S6A). Further analysis revealed that both CON and DKO neonatal hearts utilized various similar inflammatory and repair programs, however, DKO neonates upregulated a wider range of differentially expressed genes (DEGs) and the majority of those genes were related to pro-inflammatory programs such as various type-1 interferon-related genes (Figure S6B-D). The upregulation of these genes have been found by others to greatly increase the likelihood of cardiac failure after myocardial infarction 46. The analysis was further augmented with an analysis of the broad based-functions that would be impacted based on the DEGs confirming the deterioration in heart function and loss of cardiomyocyte population (Table S2).

### Loss of IL-4 and IL-13 impairs cardiac regeneration in a STAT6-dependent manner

Given that IL-4 and IL-13 signal across the STAT6 pathway, we sought to determine whether STAT6 acted as the primary driver of downstream effectors controlled by IL-4 and IL-13. Both IL-4 and IL-13 share a heterodimeric receptor, IL-4Rα, which signal to the STAT6 pathway 47. We thus utilized a small molecule inhibitor, AS1517499, to inhibit the phosphorylation of STAT6 after injury, classed as S6i sham and injured.

**Figure 5:**
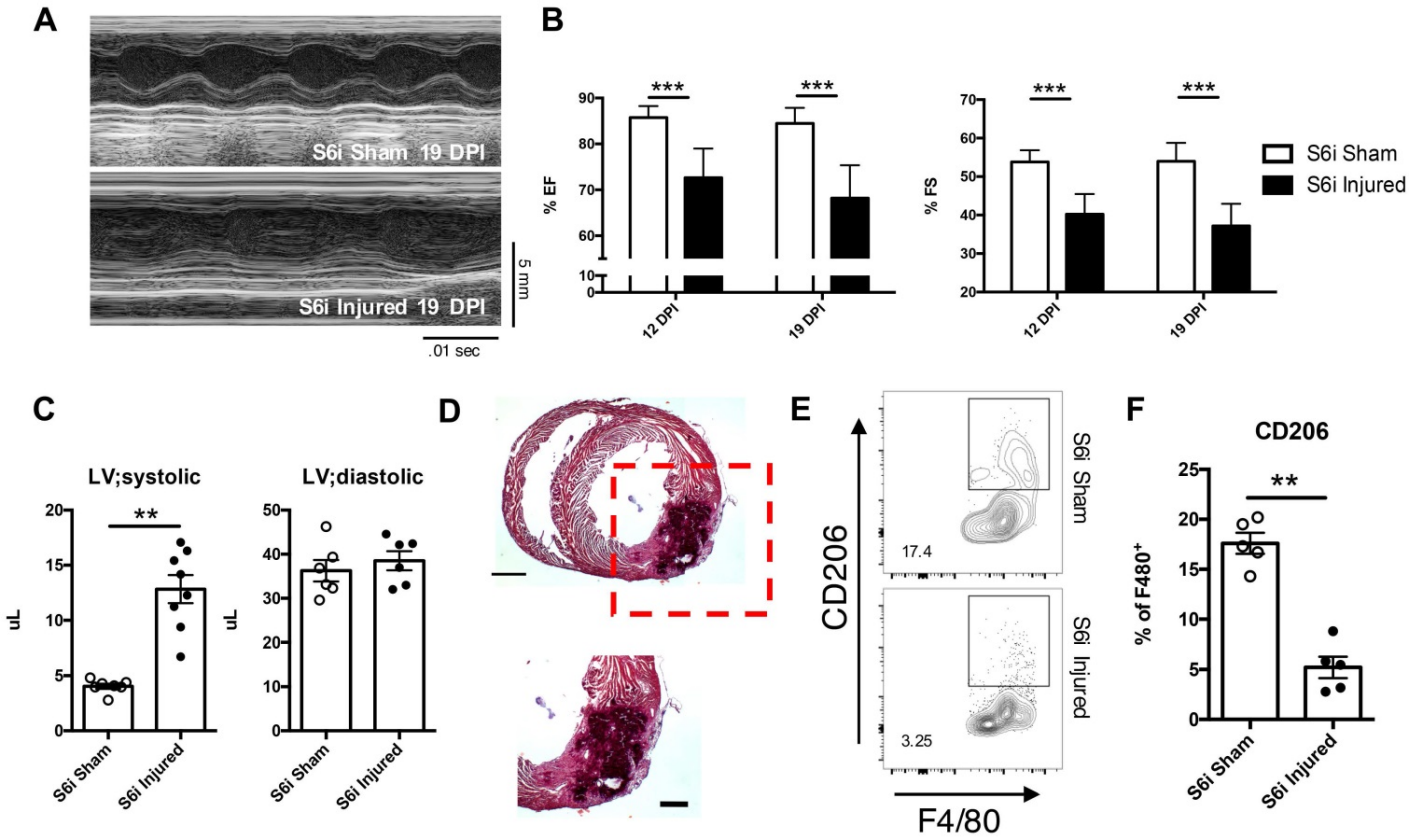
** Use of STAT6 small molecular inhibitor recapitulates cardiac regenerative defect observed in DKO injured neonatal hearts.** AS1517499 STAT6 inhibitor (S6i) injected via peritoneal injection at time of injury and subsequent days afterwards until tissue harvest. *(A)* Representative echocardiogram at 19 DPI with* (B)* Ejection fraction (EF) and fractional shortening (FS) at 12 and 19 DPI S6i sham and injured (n = 8-9). *(C)* Ventricular volume of both systole (LV; systolic) and diastole (LV; diastolic) (n = 8-9). *(D)* Representative hematoxylin & eosin staining of injured STAT6i treated heart micrographs at 12 DPI (n = 5). Scale Bar: 1 mm, 200 μm (Red inset).* (E)* Representative flow graph of alternatively activated macrophages. *(F)* Proportion of macrophages that are CD206^+^ at 2 DPI of S6i sham and injured neonatal hearts (n = 5-6). Data are presented as mean±SEM. *P < 0.05, **P < 0.01 by two-way ANOVA followed by Sidak's multiple comparisons test for groups and unpaired student's *t*-test for paired analysis.

Inhibition of STAT6 signaling resulted in similar impairment of cardiac function and regeneration observed in DKO injured hearts. Specifically, use of the STAT6 inhibitor in injured neonatal hearts resulted in similar reductions in both ejection fraction and fractional shortening similar in magnitude to DKOs (Figure 5A and B). An increase in the left ventricular systolic volume was also observed, like that observed in injured DKOs (Figure 5C). Histological analysis revealed sections of tissue of the myocardial wall with multicellular infiltration similar to injured DKO neonatal tissue (Figure 5D). Inhibition of STAT6 phosphorylation also resulted in a reduction of anti-inflammatory immune populations observed in CON injured neonatal heart tissue. (Figure 5E and F). While there were no observed differences in CD4^+^ T-cell populations, an increase in CD8^+^ T-cells was observed, reaffirming the role of IL-4 in repressing CD8^+^ T-cell populations (Figure S7D-F). Most interestingly, there was an expansion of Ly6C^LO^ monocytes in the injured S6i treated neonate, suggesting an expansion of Ly6C^LO^ monocytes is insufficient to functionally rescue cardiac deterioration after injury (Figure S7G). The observations in immune populations after STAT6 inhibition and those observed after treatment of IL-4 and IL-13 in DKO injured hearts showed that all groups required the expansion of alternatively activated macrophages for functional recovery. This led us to question whether IL-4 and IL-13 signaling specifically in the macrophage population was necessary for successful neonatal cardiac regeneration.

### Myeloid IL-4Rα signaling is required to drive anti-inflammatory macrophage populations conducive for the resolution of ischemic cardiac injury

**Figure 6:**
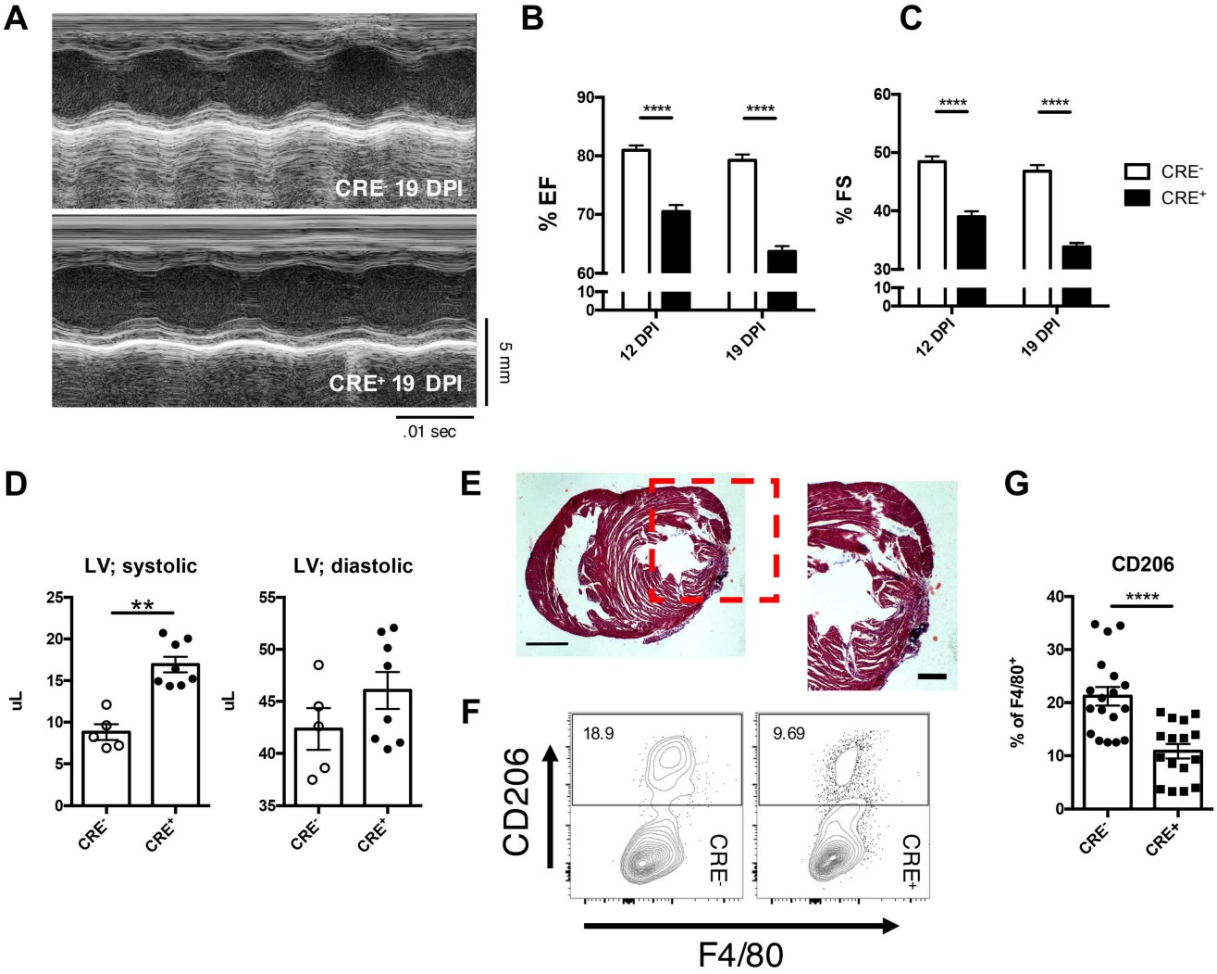
** Loss of IL-4Rα signaling in myeloid cells sufficient to impair cardiac repair from loss of alternatively activated macrophages.** *IL-4Rαff* (CRE^-^) and *IL-4Rα^ff^Lyz2^CRE^* (CRE^+^) neonatal mice were injured at P2. *(A)* Representative echocardiographs of echocardiography performed at 12 and 19 DPI to determine *(B)* Ejection fraction and *(C)* Fractional shortening (n = 8-10). *(D)* Systolic (LV; systolic) and diastolic (LV; diastolic) left ventricular mass was determined with echocardiography at 19 DPI between injured CRE^-^ and CRE^+^ P2 neonates (n = 5-7). *(E)* Representative hematoxylin & eosin staining of injured CRE^+^ heart micrographs at 12 DPI (n = 5). Scale Bar: 1 mm, 200 μm (Red inset).* (F)* Representative plot of flow cytometric analysis performed on 2DPI hearts examining alternatively activated macrophage populations with *(G)* corresponding quantitative analysis of CD206+ macrophage populations (n = 16-18). Data are presented as mean±SEM. * P < 0.05, **P < 0.01. by one-way ANOVA followed by Sidak's multiple comparisons test for groups and unpaired student's *t*-test for paired analysis.

As IL-4 and IL-13 are capable of initiating the polarization of alternatively activated macrophages, and that both IL-4 and IL-13 utilize the receptor, IL-4Rα, for downstream signaling, we examined the role of IL-4 and IL-13 in the myeloid compartment with a *IL-4Rα^ff^Lyz2^CRE^* mouse (CRE^+^) 6, 28, 29, 41, 48. Functional analysis of CRE^+^ mice after LAD ligation indicated an impairment in ejection fraction and fractional shortening (Figure 6A-C) in mice lacking IL-4Rα in the myeloid compartment compared to the CRE^-^ control injured mice. Additionally, CRE^+^ neonatal hearts exhibited similar systolic dysfunction previously observed in DKO neonatal injured hearts and similar histological patterns as those observed in DKO and STAT6 inhibitor treated neonatal hearts (Figure 6D & E). Analysis of macrophage populations indicated approximately a 50% reduction in CD206^+^ alternatively activated macrophages compared to control neonatal injured hearts (Figure 6E and F). Interestingly, various other immune subsets such as T-cells and neutrophils did not significantly alter populations in CRE^+^ neonatal injured hearts compared to control (Figure S8). Monocyte populations, however, were negatively impacted indicating a non-STAT6 mediated effect of alternatively activated macrophages on monocyte populations (Figure S8G).

### Transcriptomic analysis reveal a similar increase in negative factors of cardiac regeneration in myeloid specific IL-4Rα knockout hearts after injury as DKO hearts

In comparison to DKO injured neonatal hearts, we examined how loss of IL-4/IL-13 signaling in the myeloid compartment would alter the transcriptional landscape given similar impairment in cardiac regeneration observed in the DKO. Principal component analysis of bulk tissue sequencing revealed clear groupings between experimental groups of CRE^-^ and CRE^+^ uninjured and injured samples (Figure S9A). Moreover, an examination of differentially expressed genes in relation to IPA canonical pathway analysis indicates that loss of IL-4Rα in the myeloid compartment resulted in an increase in interferon-related genes and harmful metabolite by-products via oxidative phosphorylation similar to those observed in the DKO sequencing data analysis (Figure S9B-E, Table S2). Altogether, these findings suggest that IL-4 and IL-13 promote a conducive response to cardiac injury through the polarization of alternatively activated macrophage populations through IL-4Rα in a STAT6-dependent manner.

## Discussion

Although studies have revealed a dissimilarity between neonatal and adult immune responses after cardiac injury, the mechanisms that govern these differences remain poorly understood 7, 49. Here, we report that the basal immature, anti-inflammatory state of the immune system in the neonate directly contributes to its ability to regenerate cardiac tissue. Specifically, the role of the type 2 cytokines, IL-4 and IL-13, in maintaining a T_H_2-skewed immune profile observed in the neonatal heart, which to the best of our knowledge has never been shown previously. These findings reinforce the importance of the state of the cardiac niche on regeneration, and the seminal role of both IL-4 and IL-13 in promoting populations of cells conducive for regeneration before and after tissue damage.

The different responses and results between neonates and adult hearts after injury leave many questions to be answered. Our systematic characterization of the role of IL-4 and IL-13 on the immune population, both prior and after injury in the neonate, reveals profound shifts in the makeup of immune cells over the first days of life after birth. These changes in immune populations greatly alter both the variety of cellular players in the cardiac niche and the overall starting material to utilize after injury. Previous studies have pointed at the lack of functionality in adult cardiomyocytes to expand after injury as a significant factor for the failure of adult mammalian hearts to regenerate following damage 4. However, our studies coupled with others regarding the profile of the neonatal cardiac niche, reveal that even with cardiomyocytes, the lack of an appropriate microenvironment conducive for regeneration will result in the deteriorated functional capabilities of the heart and failure to resolve injury completely 6. Furthermore, our study further reinforces the concept that there are “keystone” populations of cells within the niche that are indispensable to the success of the regenerative process. Identification of the cell types signaled by IL-4 and IL-13, and the examination of the downstream signaling pathways revealed that regardless of the experimental inputs, the reduction of alternatively activated macrophages corresponded to the reduced capability of the heart to regenerate. While other groups have shown that complete abolishment of M2 macrophages resulted in mortality in adults following injury, our study showed the upstream role of IL-4 and the IL-4Rα in the myeloid compartment to facilitate the polarization and promotion of neonatal M2 macrophages after injury 8.

Analysis of the transcriptome in both *IL-4^-/-^/IL-13^-/-^* and *IL-4Rα^ff^Lyz2^CRE^* injured mice provided supporting results of the negative effects on cardiac regeneration when there is a deficit of anti-inflammatory immune cells dominates in the heart. Bulk sequencing analysis revealed an increase in pro-inflammatory signals such as type-1 interferon signals. Prior studies in humans and in mice indicated that an increase in type-1 interferon signals from pro-inflammatory cardiac macrophages resulted in deterioration of heart function, possibly leading to heart failure 46. In conjunction to the increase in pro-inflammatory programs that provoke further damage to cardiac tissue, loss of IL-4/IL-13 and alternatively activated macrophages led to variety of mitochondrial and metabolic dysfunction in the heart after injury. Other key programs such as miRNA-133a were found to be downregulated in both *IL-4^-/-^/IL-13^-/-^* and *IL-4Rα^ff^Lyz2^CRE^* after injury. miRNA-133a is known to regulate cardiomyocyte proliferation and cardiac development 50. Interestingly, many of the differentially expressed genes in both sets were predicted genes shown to be ribosomal in nature such as GM15772 and GM10736. These genes coupled with the downregulation of mRNA stabilization protein, ELAV1, both in the transcriptomic analysis and validated by qPCR (data not shown) indicates possible dysfunction in intrinsic factors regulating cellular machinery, leading to impairment in cardiac regeneration 51.

Our findings show that IL-4 and IL-13, cytokines that actively promote neonatal T_H_2 preference to maintain an immune-privileged state between mother and child, signals macrophage populations in the neonatal heart through IL-4Rα to suppress pro-inflammatory programs that are antithetical to cardiac repair. Our study poses interesting questions regarding how neonatal development of systems required for homeostasis interacts with the programs governing the regeneration of tissue. A variety of studies have shown that significant metabolic changes occur during the early stages of neonatal and perinatal life, ranging from increased oxygen to alterations in hormones, both of which greatly influences the mitotic senescence of cardiomyocyte populations 52-54. It has also been shown that the skewing of T_H_2 preference in the neonate reduces over the course of aging, with an increase in pro-inflammatory populations to counteract the prevalence of infection during adulthood 49. This maturation of the neonatal immune system, to some degree, chronologically tracks the loss of cardiac regeneration in mammals 55, 56. Interestingly, whether the regeneration of other tissue types in the neonate are similarly affected from the changes in niche composition is not completely understood. Finally, our study poses the translational question of whether changes in the extrinsic factors within the cardiac niche of patients with heart damage such as myocardial infarction can improve overall patient outcomes.

As our study focused primarily on the role of neonatal immunity on the outcome of cardiac function after injury, future work will examine the role of both IL-4 and IL-13 on other cellular populations in the cardiac niche such as the cardiomyocyte population itself, as cardiomyocytes retain the receptor for both IL-4 and IL-13 signaling.

## Conclusion

Our examination of neonatal immune responses after cardiac injury reveal that successful resolution of injury is due, in part, from the repression of pro-inflammatory responses and the promotion of reparative anti-inflammatory macrophage populations driven by IL-4 and IL-13. In conclusion, our current work provides evidence of a direct relationship between the repressed neonatal immune environment and successful regeneration in cardiac tissue, possibly revealing new avenues of cardiac treatment in adults.

## Supplementary Material

Supplementary figures, tables, and methods.
