# Supplementary material for: Type 2 innate immunity drives distinct neonatal immune profile conducive for heart regeneration
Source: Theranostics. 2022 Jan 1;12(3):1161–72. doi: 10.7150/thno.67515 (PMC8771554; doi:10.7150/thno.67515)
Supplement: Supplementary file 1 — Supplementary figures, tables, and methods. [file thnov12p1161s1.pdf]

Supplementary Data

Type 2 innate immunity drives distinct neonatal immune profile conducive for heart regeneration

Short title: IL-4 and IL-13 are required for cardiac regeneration

Francis M. Chen[1*], Joyce KY Tse[1], Leigang Jin[2,3], Chui Yiu Bamboo Chook[1], Fung Ping Leung[1], Gary Tse[4,5], Connie W. Woo[2,3], Aimin Xu[2,3,6], Ajay Chawla[7], Xiao Yu Tian[8], Ting-Fung Chan[1,9], Wing Tak Wong[1,9*]

[1] School of Life Sciences, Faculty of Science, The Chinese University of Hong Kong, Hong Kong, China;

[2] State Key Laboratory of Pharmaceutical Biotechnology, The University of Hong Kong, Hong Kong, China;

[3] Department of Pharmacology and Pharmacy, The University of Hong Kong, Hong Kong, China;

[4] Tianjin Key Laboratory of Ionic-Molecular Function of Cardiovascular Disease, Department of Cardiology, Tianjin Institute of Cardiology, Second Hospital of Tianjin Medical University, Tianjin, China;

[5] Kent and Medway Medical School, Canterbury, United Kingdom;

[6] Department of Medicine, Li Ka Shing Faculty of Medicine, The University of Hong Kong, Hong Kong, China;

[7] Merck Research Labs, SAF-803, South San Francisco, CA, USA;

[8] School of Biomedical Sciences, The Chinese University of Hong Kong, Hong Kong, China

[9] State Key Laboratory of Agrobiotechnology, The Chinese University of Hong Kong, Hong Kong, China.

[*] Co-Corresponding Author

Supplemental Table 1.

Major Resources Table

Animals (in vivo studies)

Species	Vendor or Source	Background Strain	Sex
BALB/c	University Laboratory Animal Services Center (The Chinese University of Hong Kong – LASEC)	BALB/c	M & F
$IL\text{-}4^{-/-}/IL\text{-}13^{-/-}$	Self-bred after obtaining from University of California, San Francisco – Ajay Chawla Laboratory	BALB/c	M & F
$IL\text{-}4Ra^{ff}$	Self-bred after obtaining from University of California, San Francisco – Ajay Chawla Laboratory	C57/Bl6	M & F
$IL\text{-}4Ra^{ff}Lyz2^{CRE}$	Self-bred after obtaining from University of California, San Francisco – Ajay Chawla Laboratory	C57/Bl6	M & F

Genetically Modified Animals

	Species	Vendor or Source	Background Strain	Other Information
**Parent – Male & Female**	*$IL\text{-}4^{-/-}/IL\text{-}13^{-/-}$*	Self-bred after obtaining from University of California, San Francisco – Ajay Chawla Laboratory	BALB/c	After transfer to present facility (CUHK-LASEC), was backcrossed to Balb/C mice for a minimum of 3 generations at CUHK - LASEC
**Parent - Male & Female**	*$IL\text{-}4Ra^{ff}$*	Self-bred after obtaining from University of California, San Francisco – Ajay Chawla Laboratory	C57/Bl6	After transfer to present facility (CUHK-LASEC), was backcrossed to C57/Bl6 mice for a minimum of 3 generations at CUHK-LASEC
**Parent – Male & Female**	*$IL\text{-}4Ra^{ff}Lyz2^{CRE}$*	Self-bred after obtaining from University of California, San Francisco – Ajay Chawla Laboratory	C57/Bl6	After transfer to present facility (CUHK-LASEC), was backcrossed to C57/Bl6 mice for a minimum of 3 generations at CUHK-LASEC

Antibodies

Target antigen	Vendor or Source	Conjugated to:	Working concentration	Clone
CD45	Biolegend #103115	APC/Cy7	0.2 ug/mL	Clone 30-F11
CD45	Biolegend #103111	APC	0.2 ug/mL	Clone GK1.5
CD3-e	Biolegend #100305	FITC	0.2 ug/mL	Clone 146-2C11
CD8a	Biolegend #100721	PE/Cy7	0.2 ug/mL	Clone 53.67
F4/80	Biolegend #123113	PE/Cy7	0.2 ug/mL	Clone BM8
F4/80	Biolegend #123125	PerCP	0.2 ug/mL	Clone BM8
CD11b	Biolegend #101229	PerCP	0.2 ug/mL	Clone M1/70
CD11b	Biolegend #101211	APC	0.2 ug/mL	Clone M1/70
Ly6C	Biolegend #128027	PerCP	0.2 ug/mL	Clone HK1.4
CD11c	Biolegend #117305	FITC	0.2 ug/mL	Clone N418
CD206	Biolegend #141703	FITC	0.2 ug/mL	Clone C068C2
Ly6G	Biolegend #127605	FITC	0.2 ug/mL	Clone 1A8
Ly6G	Biolegend #127611	PacBlue	0.2 ug/mL	Clone 1A8
IFNgamma	Biolegend #505817	PacBlue	0.2 ug/mL	Clone XMG1.2
IL-4	Biolegend #504103	PE	0.2 ug/mL	Clone 11B11
CD16/32	Biolegend #101325		0.2 ug/mL	Clone 93
STAT6	Cell Signaling #9362	Rab	1:1000	D3H4
p-STAT6	Cell Signaling #56554S	Rab	1:1000	Y641
AKT	Cell Signaling #4691	Rab	1:1000	C67E7
p-AKT	Cell Signaling #4060	Rab	1:1000	S473
ERK1/2	Cell Signaling #8544	Rab	1:1000	137F5
p-ERK1/2	Cell Signaling #4370	Rab	1:1000	T202/Y204
GAPDH	Boster Biological Tech #M00227-6	Rab	1:1000	GAPDH-71.1
Goat Anti-Rab	Invitrogen #A277036	HRP	1:2000	
Live/Dead (Aqua)	Invitrogen #L34965		1:1000	
CD206	Abcam #ab64693		1:100	
Donkey Anti-Rab	Jackson Immuno Research #111-585-003	594-conjugated	1:500	
DAPI	Abcam #ab228549		1:1000	
Hematoxylin	Sigma-Aldrich #HHS16-500mL			
Eosin Y	Sigma-Aldrich #318906-500mL			

Data & Code Availability

Description	Source / Repository	Persistent ID / URL
PRJNA730354	NCBI BIOSAMPLE	https://www.ncbi.nlm.nih.gov/sra/PRJNA730354 NOTE: link will open at time of publication

Supplemental Table 2.

Categories	Diseases or Functions Annotation	p-value
Cardiac Damage	Injury of left ventricle	1.05E-03
Cardiac Enlargment	Hypertrophy of heart cells	8.59E-02
Cardiac Infarction	Myocardial infarction	8.97E-02
Cardiac Necrosis/Cell Death	Apoptosis of cardiomyocytes	1.20E-01

IPA analysis of bulk tissue sequenced CON and DKO samples indicating most likely disease or functions annotated from DEG (differentially expressed gene) comparisons.

Supplemental Table 3.

Categories	Diseases or Functions Annotation	p-value
Cardiac Necrosis/Cell Death	Apoptosis of heart cells	1.08E-03
Cardiac Necrosis/Cell Death	Apoptosis of cardiomyocytes	2.69E-03
Cardiac Thrombosis	Thrombosis of atrium	3.41E-03
Cardiac dilation, Cardiac Enlargment	Dilated Cardiomyopathy	4.42E-03
Cardiac Dysfunction	Left Ventricular dysfunction	1.48E-02
Cardiac Inflammation	Inflammation of heart	1.40E-02
Cardiac Enlargment	Pressure overload hypertrophy	1.61E-02
Cardiac Arteriopathy, Cardiac Fibrosis	Fibrosis of coronary artery	1.61E-02
Cardiac Anteripathy	Disorder of coronary artery	1.65E-02
Cardiac Enlargment	Enlargment of heart chamber	1.65E-02
Cardiac Enlargment	Enlargment of heart	1.66E-02
Cardiac Fibrosis	Fibrosis of heart	1.66E-02
Cardiac Fibrosis	Fibrosis of heart ventricle	1.75E-02
Cardiac Stenosis	Stenosis of pulmonary valve	2.00E-02
Cardiac Damage	Rupture of heart	2.66E-02
Cardiac Dysfunction, Cardiac Enlargment	Hypertrophy of heart apex	2.91E-02

IPA analysis of bulk tissue sequenced CRE^- and CRE^+ samples indicating most likely disease or functions annotated from DEG (differentially expressed gene) comparisons.

SUPPLEMENTAL FIGURE LEGENDS

Supplemental Figure 1: Flow Plot Schematic

A, Gating strategy utilized for flow cytometric analysis.

Supplemental Figure 2. IL-4/IL-13 maintains neonatal immune landscape distinct to adult immune populations in the heart. P2 (2 days after birth) neonatal and adult (10 week old) *BALB/c* control (CON) and $IL\text{-}4^{-/-}/IL\text{-}13^{-/-}$ (DKO) hearts were isolated to determine immune populations using flow cytometry. **A,** Cells/mg of $CD45^{+}$ hematopoietic cells between neonatal and adult CON and DKO hearts. **B,** CD4/CD8 Ratios (n = 5-8), $CD4^{+}$, $CD8^{+}$ populations (n = 5-8). Between neonatal and adult groups: $p < 0.05$. **C,** T_H1 and T_H2 populations of $CD4^{+}$ T-cells (n=5-6); [**P < 0.01 , T_H1-all groups significant except DKO neo vs DKO adult]. **D,** $Ly6C^{HI/LO/MID}$ monocyte populations (n = 6-12). **E,** $Ly6G^{+}$ neutrophil populations (n = 6-12). **F,** $F4/80^{+}$ macrophage populations (n = 6-12). **G,** Alternatively activated $F4/80^{+}CD206^{+}$ macrophage populations (n = 6-8). Data are presented as mean±SEM. *P < 0.05, **P < 0.01. by two-way ANOVA followed by Sidak's multiple comparisons test.

Supplemental Figure 3: Time course examination of immune cell populations indicate an increased flux of immune cell response at 2 DPI.

An examination of key immune populations was examined in both CON and DKO neonatal mice injured 2 days after birth. **A,** Absolute cell numbers of $CD45^{+}$ hematopoietic stem cells (n = 5-6). **B,** Absolute cell numbers of $F480^{+}$ macrophage populations (n = 5-6), C) Proportion of alternative activated $CD206^{+}$ macrophages (n = 5-6). **D,** Proportion of $Ly6G^{+}$ neutrophils (n=5-6). **E,** Absolute cell numbers of $CD3^{+}$ cells (n = 5-6). **F,** Proportion of $CD4^{+}$ (n = 5-6) and **G,** $CD8^{+}$ T-cells (n = 5-6). Data are presented as mean±SEM. *P < 0.05, **P < 0.01 by one-way-ANOVA followed by Sidak's multiple comparisons test.

Supplemental Figure 4. Loss of IL-4 and IL-13 Results in Preference for $CD8^{+}$ Cytotoxic T cell Populations After Injury

P2 neonatal hearts underwent LAD ligation and were harvested for FACs analysis at 2 DPI. **A,B,** Representative Flow Plots and summarized absolute cell numbers of $CD45^+$ hematopoietic stem cell populations (n = 16-18). **C,** Absolute cell numbers of $CD3^+$ T-cells at CON and DKO neonatal hearts at 2 DPI (n = 10-14). **D,** Representative flow plots of CD4/CD8 populations. **E,** $CD4^+$ (n = 11-12) and **F,** $CD8^+$ populations (n = 11-12). **G,** CD4/CD8 Ratio (n = 11-12). Data are presented as mean±SEM. * P < 0.05, **P < 0.01 by two-way-ANOVA followed by Sidak's multiple comparisons test.

Supplemental Figure 5: IL-4 and IL-13 have varying effects on different cell immune cell populations after injury

A, Absolute numbers of $CD45^+$ cell (n = 6-10). **B,** Absolute numbers of $CD3^+$ cell (n = 6-10). **C,** $CD4^+$ and $CD8^+$ (n = 5-9). **D,** CD4/CD8 ratio (n = 5-9). **E,** $Ly6c^{HI}$, $Ly6c^{LO}$, $Ly6c^{MID}$ monocytes (n = 5-8). after IL-4 or IL-13 treatment. Data are presented as mean±SEM, *P < 0.05, **P < 0.01 by two-way-ANOVA followed by Sidak's multiple comparisons test.

Supplemental Figure 6. Bulk RNA-seq analysis of Control and DKO neonatal hearts indicate an increase in negative effectors to cardiac regeneration. A, PCA plots of CON and DKO uninjured and injured samples. **B,** MA plot of CON and DKO injured. **C,** Volcano plot of CON and DKO injured. **D,** Heatmap of significantly differentially expressed genes (DEGs, padj < 0.05).

Supplemental Figure 7. Effects of AS1517499 STAT6 inhibitor on immune populations in the injured neonatal heart

Analysis of monocytes, macrophage, T-cell populations in the injured neonatal heart after injection of AS1517499. Absolute cell numbers of **A,** $CD45^+$ hematopoietic stem cells (n = 9-10). **B,** $CD3^+$ (n = 4-7). **C,** $F4/80^+$ macrophage populations (n = 5-6). **D,** $CD4^+$ (n = 3-4). **E,** $CD8^+$ (n = 3-4) T-cell populations with corresponding **F,** Ratios between CD4/CD8 T-cell

populations (n = 3-4). **G,** Ly6C$^{HI/MID/LO}$ monocyte populations (n = 4). Data are presented as mean±SEM. *P < 0.05, **P < 0.01 by unpaired student's *t*-test.

Supplemental Figure 8: Loss of myeloid IL-4Rα did not alter non-myeloid populations in response to injury

Flow cytometric analysis was performed on 2DPI P2 CRE^{-} and CRE^{+} neonates. **A,** Absolute number of CD45^{+} hematopoietic stem cells (n = 10-12) **B,** absolute number of CD3^{+} (n = 7-8). **C,** absolute number of F4/80^{+} macrophages (n = 7-9). **D,** CD4^{+} and CD8+ T-cell populations (n = 6-9). **E,** CD4/CD8 ratios (n = 6-9). **F,** T_H1 (IFN-γ^{+}) to T_H2 (IL-4^{+}) CD4^{+} T-cell populations (n = 6). **G,** Ly6C$^{HI/MID/LO}$ monocytes (n=6-7). **H,** Ly6G^{+} neutrophil populations (n = 6-7). *P < 0.05, **P < 0.01 by unpaired student's *t*-test.

Supplemental Figure 9: Bulk RNA-seq analysis of myeloid-specific IL-4Rα knockout reveals impaired regenerative effectors in the neonatal heart after injury. A, PCA plots of CRE- and CRE+ uninjured and injured samples. **B,** MA plot of CRE- injured vs. CRE+ injured. **C,** Volcano plot of CRE- injured vs. CRE+ injured. **D,** Heatmap of significant differentially expressed genes (DEGs, padj < 0.05).

Supplemental Figure 10: CD206 macrophage population across various neonatal heart populations. A, Cells/mg population of CD206 macrophage populations. **B,** Representative immunofluorescent micrographs of neonatal hearts injured on P2 and harvested at 2 DPI. (n = 3 per group) Scale Bar: 100 µm. Data are presented as mean±SEM. * P < 0.05, **P < 0.01. by one-way ANOVA followed by Sidak's multiple comparisons test for groups and unpaired student's *t*-test for paired analysis.

SUPPLEMENTAL FIGURE 1:

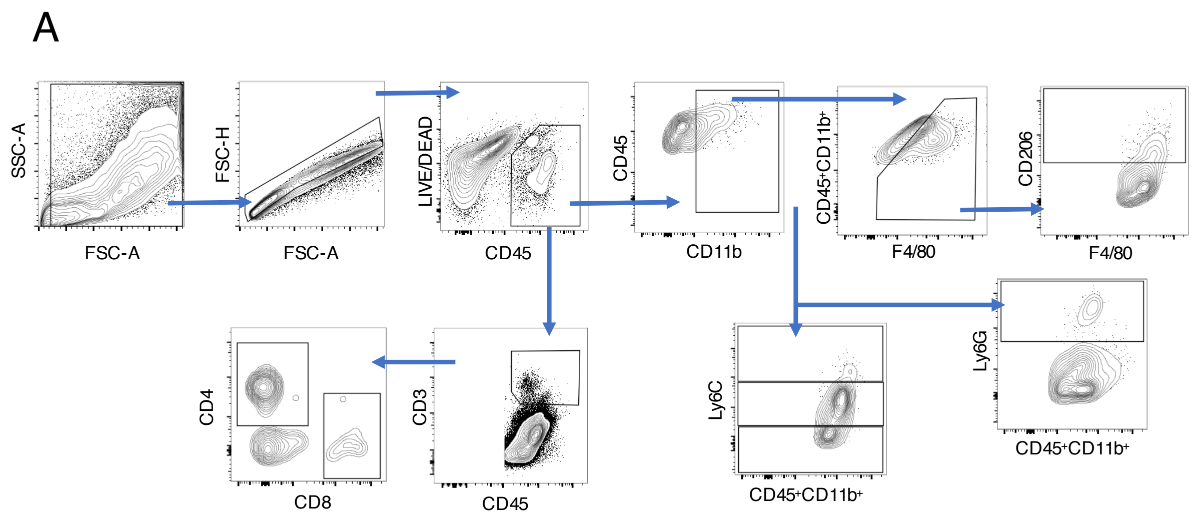

SUPPLEMENTAL FIGURE 2

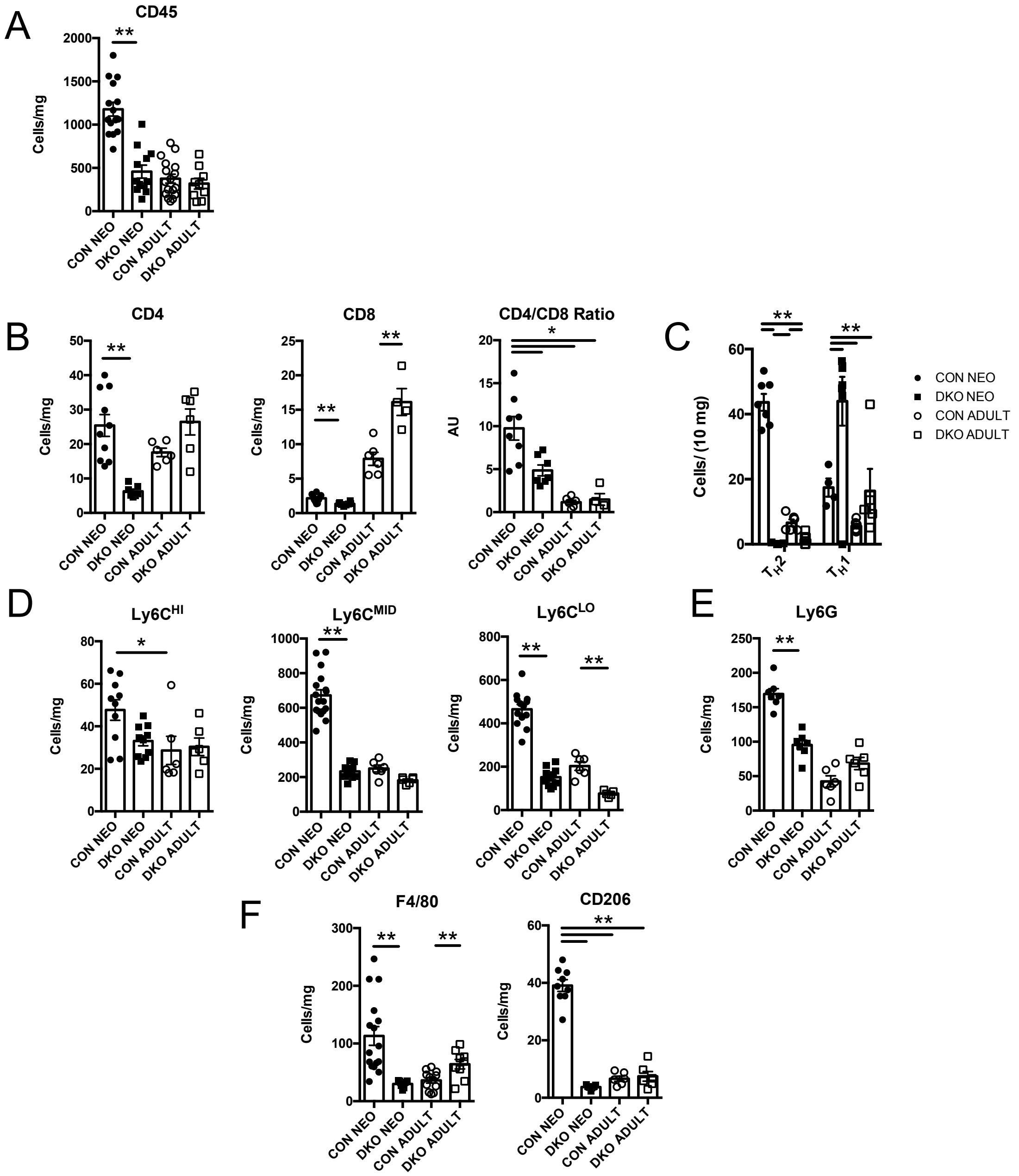

SUPPLEMENTAL FIGURE 3:

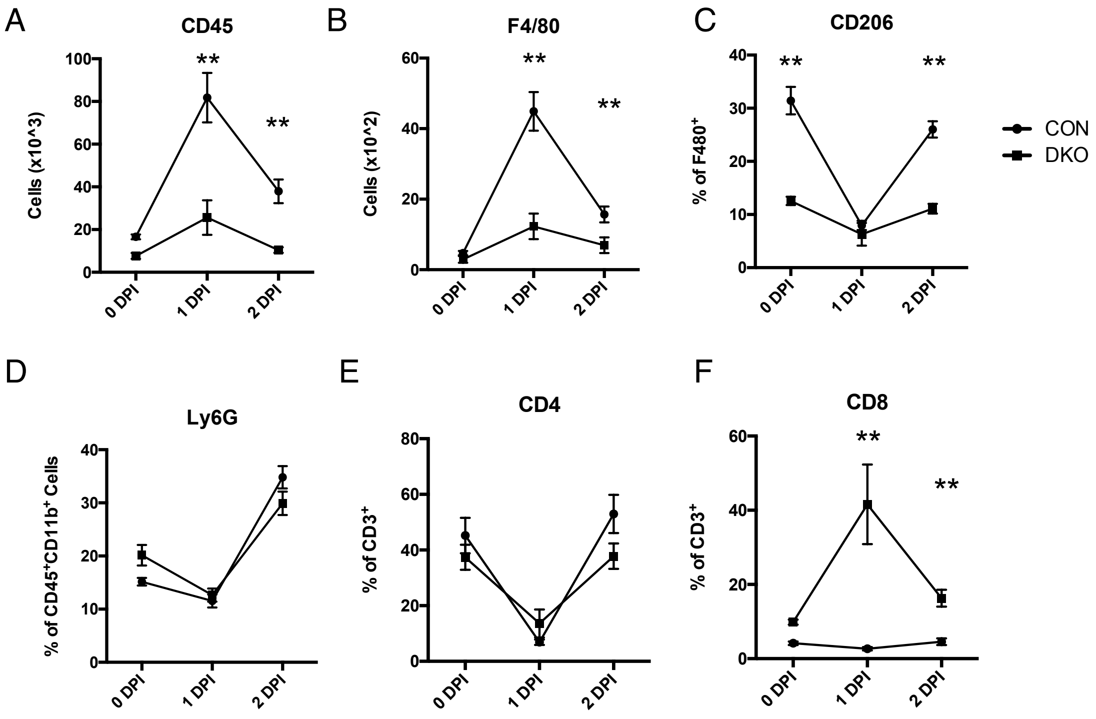

SUPPLEMENTAL FIGURE 4:

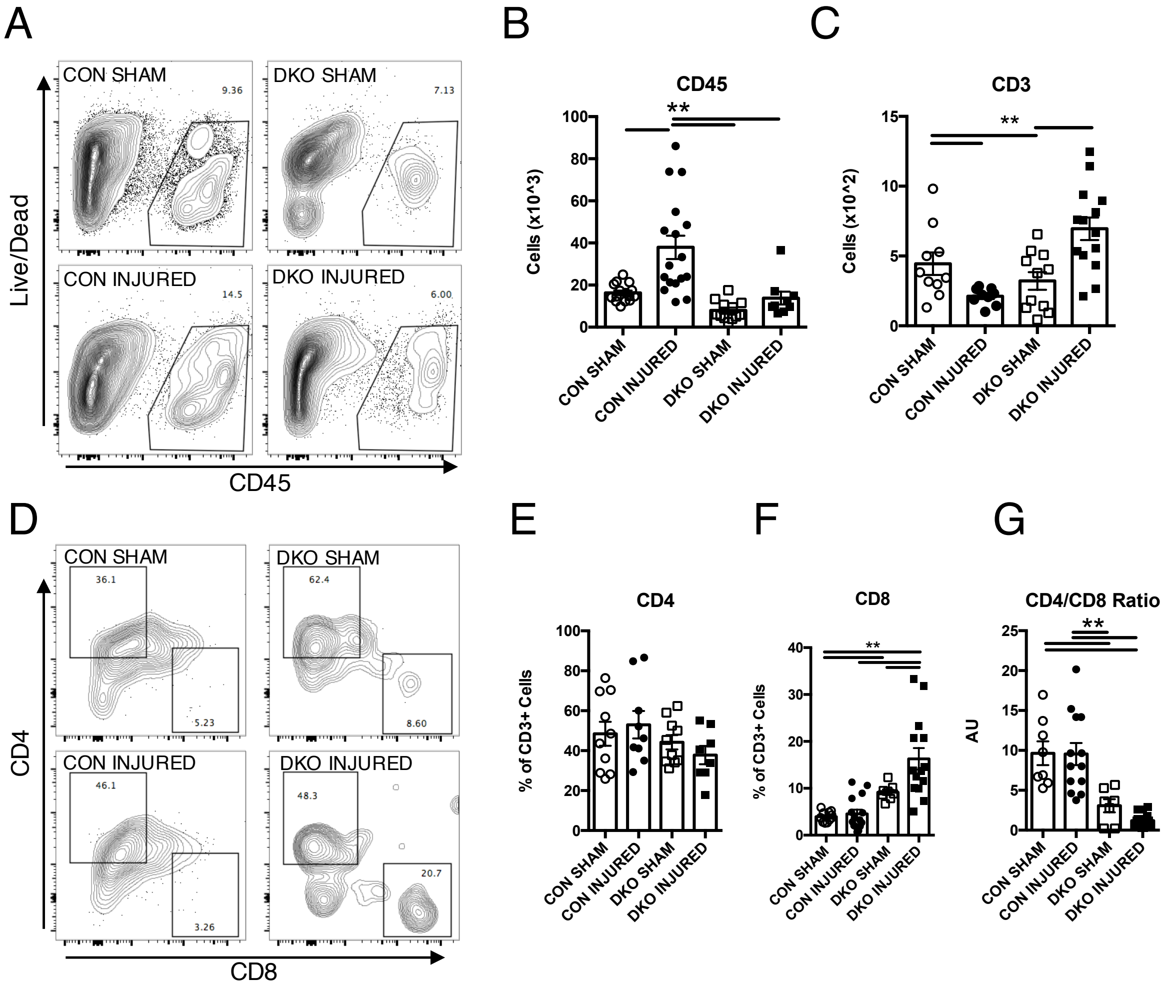

SUPPLEMENTAL FIGURE 5:

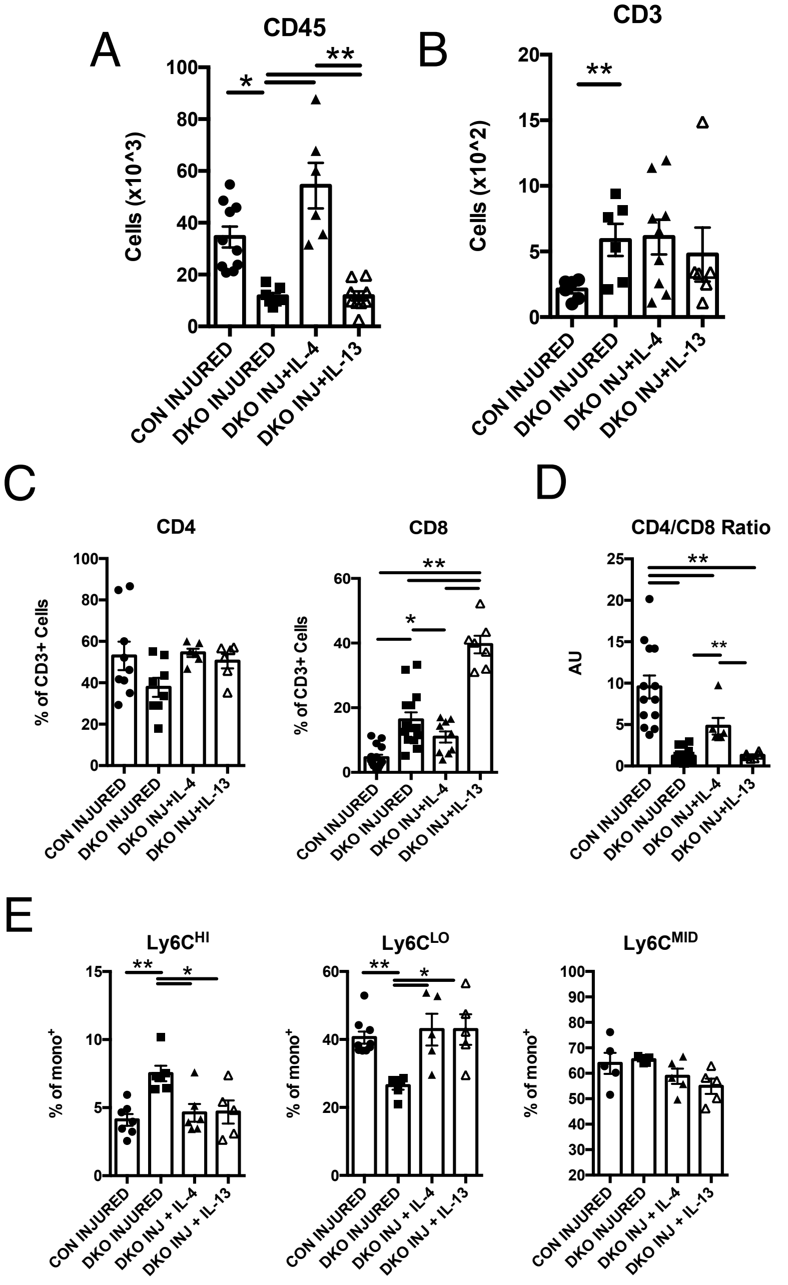

SUPPLEMENTAL FIGURE 6:

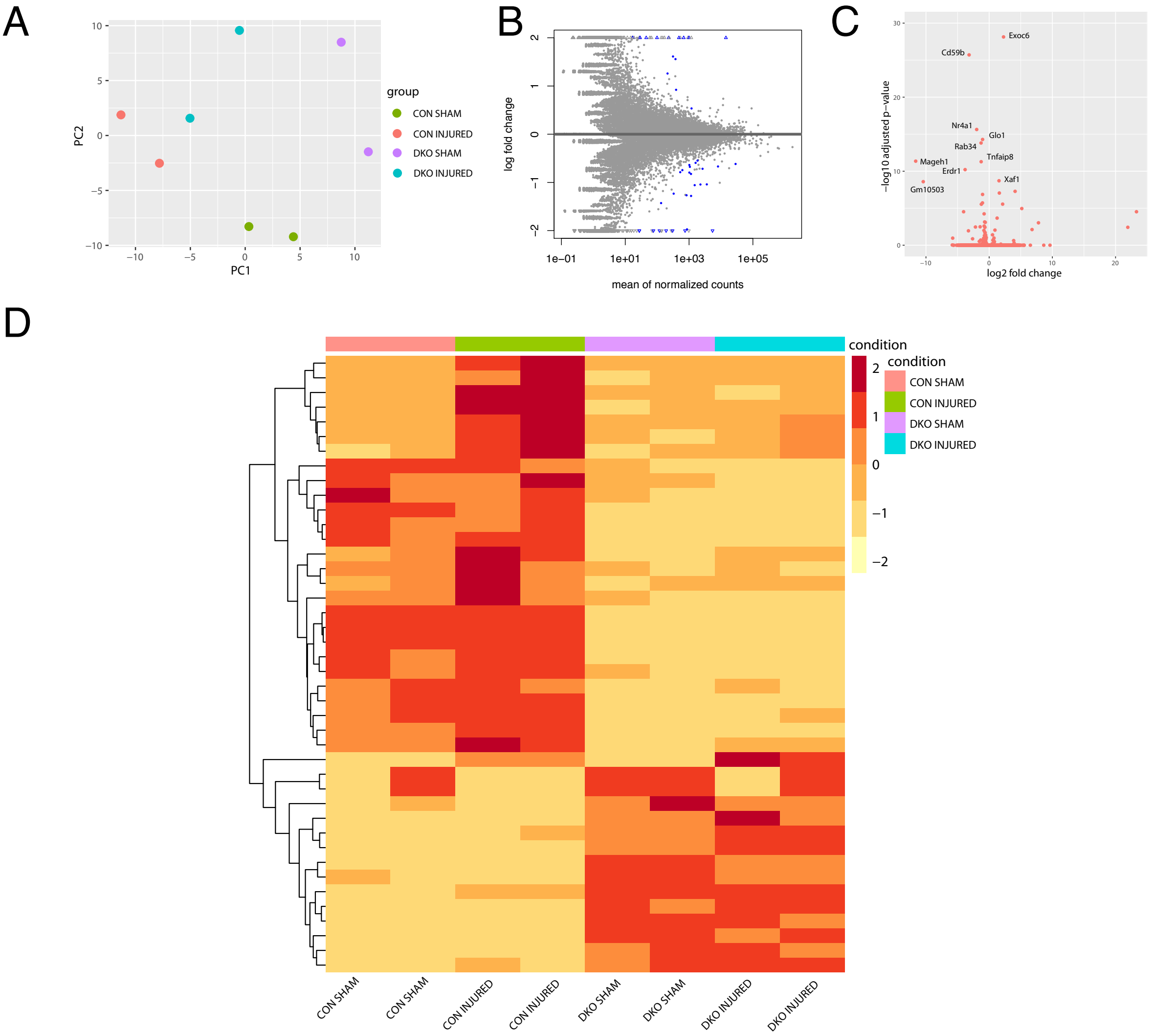

SUPPLEMENTAL FIGURE 7:

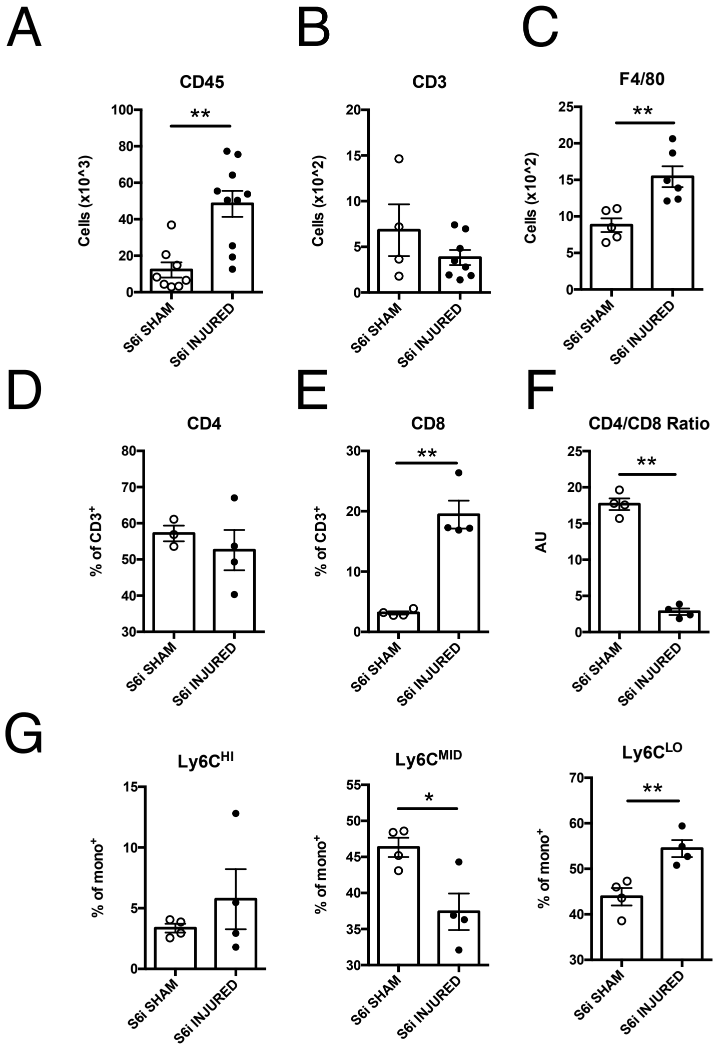

SUPPLEMENTAL FIGURE 8:

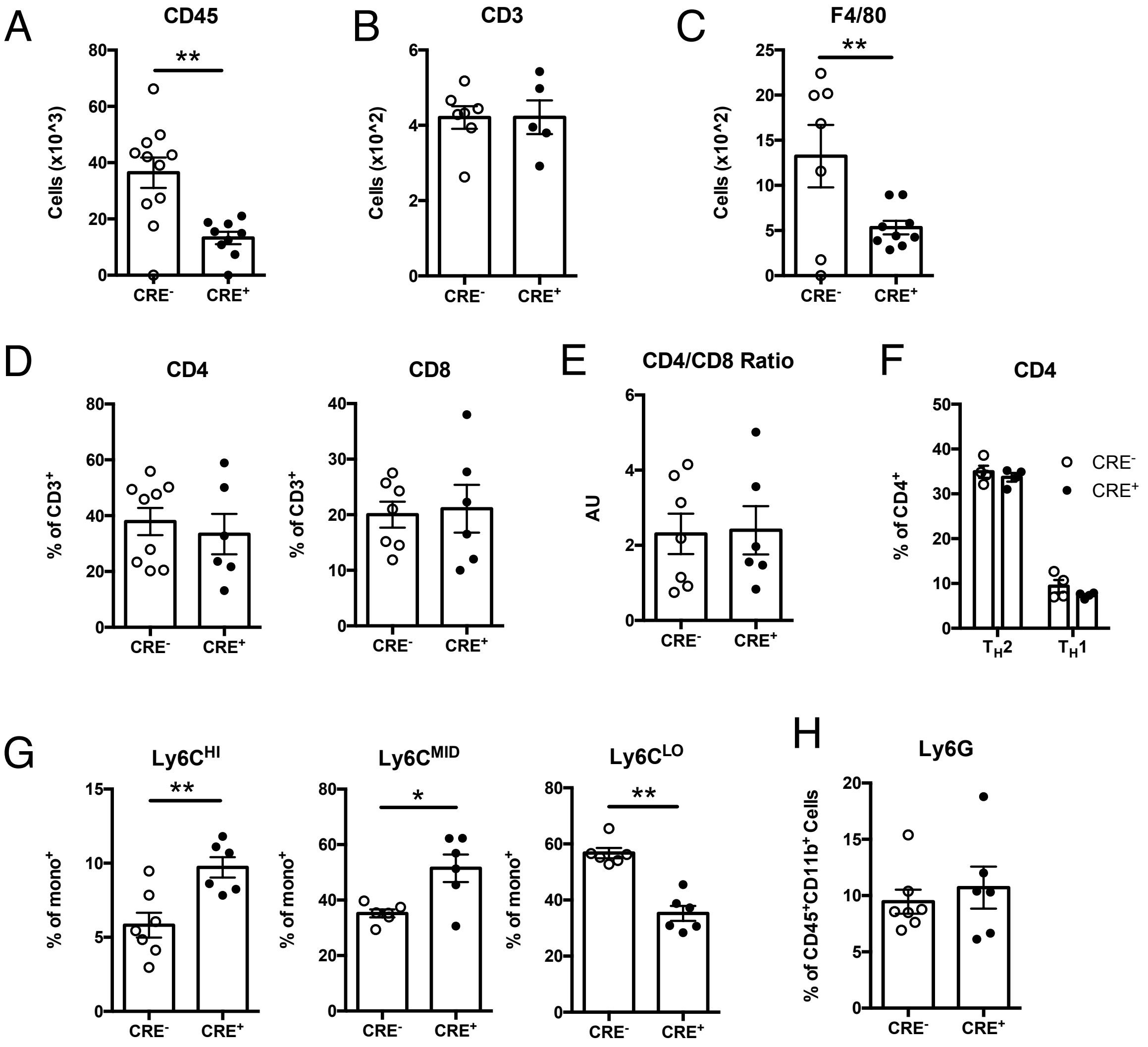

SUPPLEMENTAL FIGURE 9:

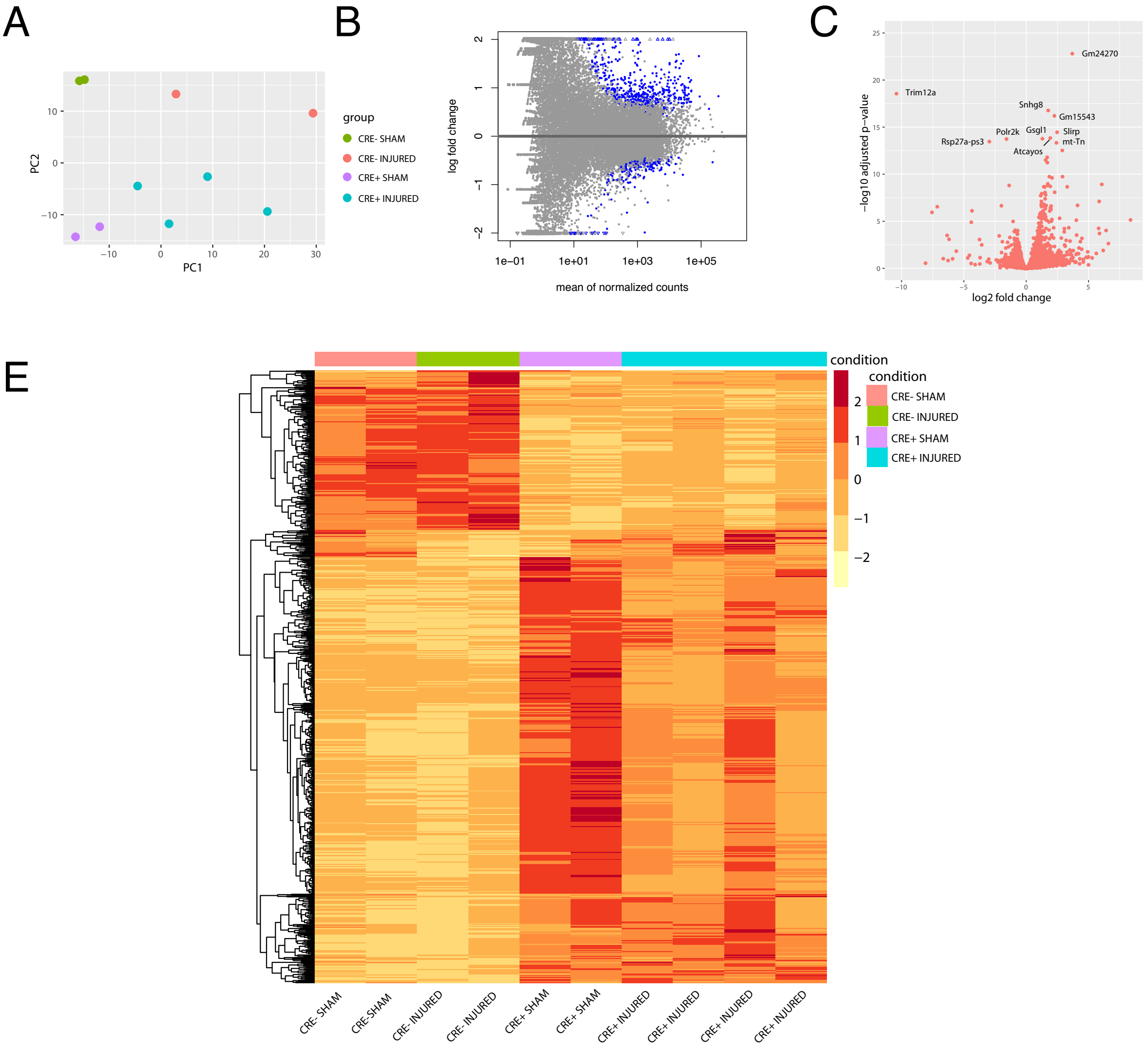

SUPPLEMENTAL FIGURE 10:

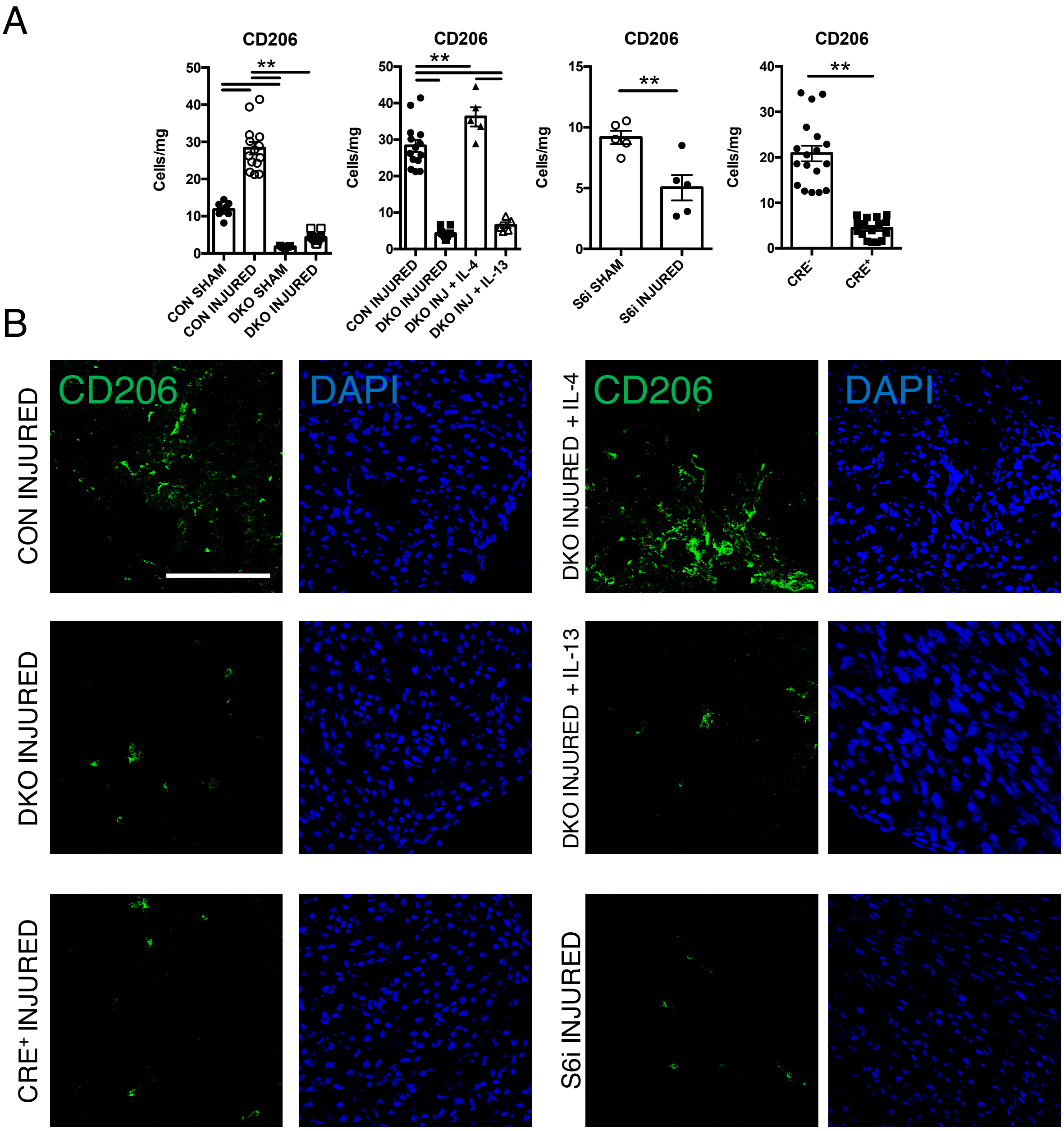

EXTENDED METHODS:

RNA-sequencing

Neonatal heart samples were isolated at 2 DPI. RNA was extracted using an RNeasy MIDI kit (Qiagen, Hilden, Germany). Library preparation and RNA sequencing were performed by Novogene (China), following Illumina's protocols for poly(A) selection and NovaSeq6000 paired-end sequencing (2 x 150 bp). Each condition (DKO uninjured, DKO injured, CRE^{-}/CRE^{+} uninjured and CRE^{-}/CRE^{+} injured) had at least two biological replicates. An average of 87 ± 8.8 million reads were obtained for each sample. FastQC (v0.11.7) was first used to check the raw sequencing data quality. The raw data were subjected to trimming of low quality reads by Trimmomatic (v0.38). Trailing sequences were removed when the quality score was less than 20, sequences with a minimum length of 50 bp after trimming were kept, and each overall sequence must have a quality score of 30. Then, the retained reads were mapped to the *Mus musculus* reference genome (GRCm38.p6) by Star (v2.6.0a), where uniquely mapped reads were at least 82%. Picard was performed to check on mapping quality and strand specificity. A count table was subsequently generated by featureCounts (v1.6.3) and was used as an input for DESeq2 (v1.30.0) to determine the differentially expressed genes (DEGs) between samples. The DEGs lists (cutoff p-value adjusted (padj) < 0.05) were used for Ingenuity Pathway Analysis (Qiagen) to determine the important signaling pathways, disease functions and molecules involved. DESeq2 was also used for the principal component analysis (PCA) plots, volcano plots, MA plots and heatmaps of DEGs. The heatmaps were generated based on the DEGs (padj < 0.05) between the CON injured and DKO injured for the CON-DKO heatmap and between the Cre^{-} injured and Cre^{+} injured for the CRE^{-}/CRE^{+} heatmap. Samples have been uploaded to NCBI with the following BioProject Accession number: PRJNA730354.

Equations used:

% Ejection Fraction: $100 \times \left(\frac{LV\ Vol;d - LV\ vol;s}{LV\ Vol;d}\right)$

% Fractional Shortening: $100 \times \left(\frac{LVID;d - LVID;s}{LVID;d}\right)$

LV Mass (uncorrected): $1.053 \times ((LVID;d + LVPW;d + IVS;d)^3 - LVID;d^3)$

LV Mass (corrected): $LV\ Mass \times 0.8$

LV Vol;d: $\frac{7}{2.4+LVID;d} \times LVID;d^3$

LV Vol;s: $\frac{7}{2.4+LVID;s} \times LVID;s^3$

Flow Cytometry and Tissue Preparation

Single cell suspensions of hearts from P2, P4, and 10 week old mice were generated immediately before analysis by flow cytometry, as previously described (1). Briefly, individual hearts were minced and digested in collagenase/DNAse solution in DMEM (125 U/mL Collagenase Type II, Worthington; 60 U/mL DNAse-1, Worthington, Lakewood, NJ) for 30 minutes at 37°C under slow agitation. Digested samples were passed through a 100-µm filter (BD), and incubated with RBC lysis buffer (Biolegend, San Diego, CA) for 5 minutes. Cells were subsequently washed with PBS (Thermo Fisher Scientific, Waltham, MA), and suspended in FACs buffer (PBS, 1% BSA, 0.1 mM EDTA; Millipore-Sigma, Burlington, MA) for staining. Cells were permeabilized with permeabilization buffer for intracellular staining. Flow cytometry analysis was done using a BD FACsverse. Sample analysis was performed using FlowJo (BD, Franklin Lakes, NJ).

Quantitative Polymerase Chain Reaction (PCR)

Heart samples were placed in Trizol (Millipore Sigma) and homogenized using a mortar and pestle in conjunction with liquid nitrogen. Briefly, RNA eluent was separated from homogenized samples in Trizol using bromochloropentane (Millipore Sigma) and subsequently precipitated using ice-cold isopropanol. Precipitated RNA was washed in 70%

ethanol before converted to cDNA using a PrimeScript 1st strand cDNA synthesis kit (Takara, Kusatsu, Shiga, Japan). cDNA synthesis was performed with Vertiti 96 well thermal cycler (BD). Real time quantitative PCR was performed using a QuantiNova SYBR green real-time PCR kit (Qiagen, Hilden, Germany). Primer sequences for transcripts examined available upon request.

Hematoxylin & Eosin

Heart samples were harvested at 12 DPI and frozen in OCT. Samples were subsequently cryosectioned using a Leica CM 1950 (Leica, Germany) at 8 μm thickness. Sectioned samples were treated with hematoxylin and eosin as previously described [2]. Samples were imaged using a Carl Zeiss PALM Inverted Microscope (Zeiss, Germany).

Immunofluorescence & Confocal Microscopy

Heart samples were harvested at 2DPI of neonatal hearts injured at P2. Samples were frozen in OCT and sectioned as mentioned previously. Sample sections were fixed with ice cold methanol, and stained with CD206 and DAPI. Samples were imaged using confocal microscopy using a Leica TCS SP8 high speed imaging system (Leica).

Statistics

All statistical analyses were performed using Prism (7.0; GraphPad Software, La Jolla, CA). Quantified data represent the findings of three or more independent experiments. Results represent mean±SEM. ANOVA were used for multiple comparisons followed by *post-hoc* tests, while the Student's *t*-test (unpaired two-tailed) were used to determine statistical significance for paired groups. Quantification of qRT-PCR transcript was normalized to mean control transcript levels set at 1. The level of significance is denoted as $p<0.05$.

EXTENDED METHODS REFERENCES:

1. Porrello ER, Mahmoud AI, Simpson E, Hill JA, Richardson JA, Olson EN, et al. Transient regenerative potential of the neonatal mouse heart. Science. 2011;331(6020):1078-80.

2. Fischer AH, Jacobson KA, Rose J, Zeller R. Cutting sections of paraffin-embedded tissues. CSH Protoc. 2008;2008:pdb prot4987.
